# Metal-Oxides- and Metal-Oxyhydroxides-Based Nanocomposites for Water Splitting: An Overview

**DOI:** 10.3390/nano13132012

**Published:** 2023-07-05

**Authors:** Tse-Wei Chen, Shen-Ming Chen, Ganesan Anushya, Ramanujam Kannan, Pitchaimani Veerakumar, Mohammed Mujahid Alam, Saranvignesh Alargarsamy, Rasu Ramachandran

**Affiliations:** 1 Department of Materials, Imperial College London, London SW7 2AZ, UK; t.chen19@imperial.ac.uk; 2 Department of Chemical Engineering and Biotechnology, National Taipei University of Technology, Taipei 10608, Taiwan; saranvignesh1507@gmail.com; 3 Department of Physics, St. Joseph College of Engineering, Chennai 602117, India; g.anushya7@gmail.com; 4 Department of Chemistry, Sri Kumara Gurupara Swamigal Arts College, Thoothukudi 628619, India; tgrk2013@gmail.com; 5 Department of Biochemistry, Saveetha Institute of Medical and Technical Sciences (SIMATS), Chennai 600077, India; 6 Research Center for Advanced Materials Science (RCAMS), King Khalid University, Abha 61413, Saudi Arabia; malm@kku.edu.sa; 7 Department of Chemistry, College of Science, King Khalid University, Abha 61413, Saudi Arabia; 8 Department of Chemistry, The Madura College, Vidya Nagar, Madurai 625011, India; ramachandran@maduracollege.edu.in

**Keywords:** metal oxyhydroxide, water splitting, oxygen evolution, hydrogen evolution

## Abstract

Water electrolysis is an important alternative technology for large-scale hydrogen production to facilitate the development of green energy technology. As such, many efforts have been devoted over the past three decades to producing novel electrocatalysis with strong electrochemical (EC) performance using inexpensive electrocatalysts. Transition metal oxyhydroxide (OxH)-based electrocatalysts have received substantial interest, and prominent results have been achieved for the hydrogen evolution reaction (HER) under alkaline conditions. Herein, the extensive research focusing on the discussion of OxH-based electrocatalysts is comprehensively highlighted. The general forms of the water-splitting mechanism are described to provide a profound understanding of the mechanism, and their scaling relation activities for OxH electrode materials are given. This paper summarizes the current developments on the EC performance of transition metal OxHs, rare metal OxHs, polymers, and MXene-supported OxH-based electrocatalysts. Additionally, an outline of the suggested HER, OER, and water-splitting processes on transition metal OxH-based electrocatalysts, their primary applications, existing problems, and their EC performance prospects are discussed. Furthermore, this review article discusses the production of energy sources from the proton and electron transfer processes. The highlighted electrocatalysts have received substantial interest to boost the synergetic electrochemical effects to improve the economy of the use of hydrogen, which is one of best ways to fulfill the global energy requirements and address environmental crises. This article also provides useful information regarding the development of OxH electrodes with a hierarchical nanostructure for the water-splitting reaction. Finally, the challenges with the reaction and perspectives for the future development of OxH are elaborated.

## 1. Introduction

The salient features of energy storage and conversion systems have received considerable interest and have been successfully applied in various applications such as traditional industries, transportation, and electric vehicles [1]. The development of green and sustainable energy storage and conversion devices is a highly essential and pressing need to meet the global energy demand. Importantly, the water-splitting process is one of the promising energy systems for the production of clean and green hydrogen energy via water electrolysis. Water electrolysis comprises two half-cell reactions that take place via an oxygen evolution reaction (OER) at the anode and a hydrogen evolution reaction (HER) at the cathode [2,3]. In the past decades, extensive research has been conducted on the development of various metal oxides and oxyhydroxide (OxHs)-based electrocatalysts due to their unique variable valence states, moderate energy density, high power density, ultrahigh energy storage capacity, and long cycle life. Owing to these characteristics, these catalysts are unique [4]. Here, we discuss energy demands and the current trends in metal-OxH-based composite materials in various EC applications including the water-splitting reaction [5], OER [6], and oxygen reduction reactions (ORR) [7]. Generally, more efficient OxH-based electrocatalysts have been synthesized using different approaches [8,9], including the sol-gel [10], template-assisted [11], microwave-assisted [12], and hydrothermal (HT) [13] methods. However, with these methods, controlling the size and shape of the electrocatalysts is challenging. Notably, EC techniques have successfully been used as a powerful tool for the fabrication of a highly efficient and stable polymer-based OxH composite [14]. Remarkably, for OxH-based electrocatalysts, their mass transport properties have been effectively enhanced in their charge-transfer process to promote their electrocatalytic activities [15,16,17]. The EC mechanism and kinetic study of the OER on a nickel-based electrode under alkaline conditions to form a thin film layer of nickel OxHs can be described as below [18]:

(1) $${Ni\left(OH\right)}_{2}+{OH}^{-}+e^{-}⇋NiOOH+H_{2}O$$

Transition metal OxH supported electrodes are considered as promising active electrode candidates: they have been successfully employed to promote the OER and oxygen reduction reaction (ORR) [19,20]. Additionally, metal-oxides- and OxHs-based electrocatalysts play an important role in a vast number of EC applications including in catalysis, as EC sensors, and as energy storage devices. They also have different optical, physiochemical, thermal, and electrical properties [21]. Liang et al. [22] paid special attention to the construction of hierarchical Ni–Fe oxyhydroxide@NiFe alloy nanowire array electrocatalyst via a magnetic-field-assisted chemical deposition method. Moreover, the constructed OER electrode exhibited the current densities of 500 and 1000 mA cm^−2^ stably over 120 h at overpotentials of only 248 mV and 258 mV respectively. 

Gamma radiolysis is an interesting and scalable route for the fabrication of graphite oxide based cobalt oxyhydroxide (GO-CoOOH) electrocatalyst. Electrostatic interaction occurs between CoOOH and GO that promotes a fast electron transfer process for ORR [23]. Considerable research effort has been devoted to the development of copper OxH (CuOOH) electrocatalysts. Thus, this electrocatalyst is the most promising candidate to significantly enhance OER and ORR activities [24]. Hefnawy et al. [25] fabricated nickel–manganese double hydroxide on reduced graphene oxide (RGO-Ni-Mn double hydroxide). Additionally, the RGO-Ni-Mn double-hydroxide electrocatalyst showed remarkable EC performance toward both anodic and cathodic reactions, i.e., the achieved ethylene glycol electrooxidation current density was 38 mA cm^−1^ at 500 mV vs. Ag/AgCl. Table 1 presents a summary of representative OxH-based electrocatalysts for water-splitting, OER, and HER [26,27,28,29,30,31,32,33,34,35]. In this regard, we focused on the recent development of OxH-based nanocomposite for the EC study of water-splitting, OER, and HER activities. Here, we discuss the history of the water-splitting and OER/HER reaction mechanisms; their relevant EC activities and key stabilities of OxH-based electrocatalysts are briefly discussed. Finally, this review article highlights beneficial information for researchers in the design of interesting and novel OxH-based supported electrocatalysts for the production of clean hydrogen energy through the water-splitting process.

## 2. OxH-Based Nanomaterials for Water Splitting

Significant process has been made in the development of eco-friendly and low-cost technology for the EC water-splitting process, which is one of the most promising approaches for the large-scale production of clean hydrogen fuel [36]. Hydrogen is promising green fuel for next-generation energy carriers due to its carbon-free and renewable characteristics during the electrolysis of water [37,38]. In this regard, the EC technique has emerged as promising for both OER and HER. One of the major obstacles to the water-splitting reaction is their required overpotential to achieve a satisfactory reaction process [39]. To date, an inexpensive precious-metal OxH-based electrocatalysts have received great attention regarding the EC performance of OER and HER under alkaline conditions [40]. For instance, Huo et al. discussed the mechanistic aspect of the typical electrolysis of a water splitting system that occurs under alkaline conditions as follows [41]:

At anode:

(2) $$4{OH}^{-}\to 2H_{2}O+O_{2}+4e^{-}$$

At cathode:

(3) $$2H_{2}O+4e^{-}\to 2H_{2}+4{OH}^{-}$$

**Scheme 1:**
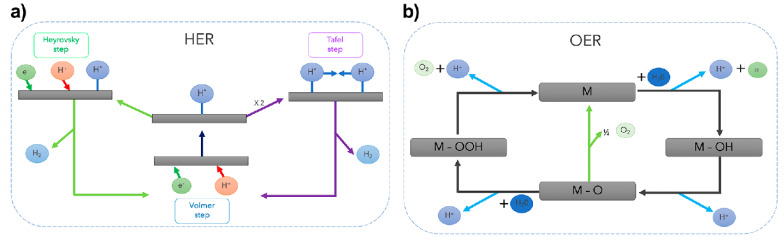
(**a**) The reaction mechanism of hydrogen evolution on an electrode surface under acidic solutions. (**b**) OER mechanism under acid conditions. The OER proceeds via the generation of an M-OOH intermediate (black line). The green line represents the direct formation of O_2_ via the interaction of M-O oxo intermediates. Copyright 2023 by the American Chemical Society [39].

In general, water molecules are abundant, so enable the sustainable production of hydrogen and oxygen through the water electrolysis process. A detailed schematic representation of the HER and OER mechanisms (Scheme 1) is necessary to understand and improve the design of electrolyzers [39].

Consequently, considering the reaction mechanisms of both OER and HER in Equations (4) and (5), the electrode potentials of these two half-reactions are related to their partial pressure of O_2_ or H_2_.

(4) $$2H_{2}O\to O_{2}+4H^{+}+4e^{-}\;(\mathrm{OER})$$

(5) $$2H^{+}+2e^{-}\to H_{2}\;(\mathrm{HER})$$

**Figure 1:**
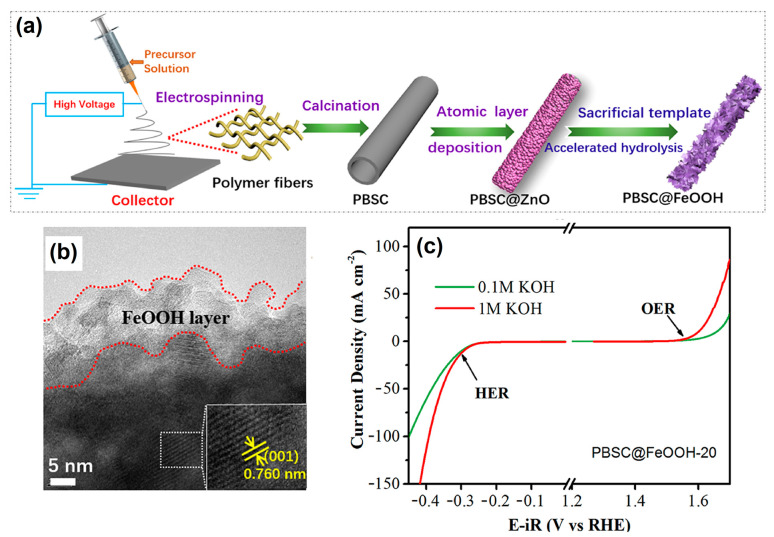
(**a**) Fabrication of integrated PBSC@FeOOH-20 catalysts with core–shell structure, (**b**) HR-TEM images of amorphous FeOOH-decorated PBSC catalyst, and (**c**) OER and HER activities of PBSC@FeOOH-20 in 0.1 M and 1 M KOH. Copyright 2018 by the American Chemical Society [46].

Currently, vanadium-based hollow nanosphere-like OxHs electrocatalysts are considered the most promising electrode for highly efficient water-splitting reactions. The electrocatalytic behavior of lepidocrocite (VOOH) was tested with oxygen saturation under alkaline conditions. Specifically, the optimized VOOH electrode cell voltage was 1.62 V at the applied current density of 10 mA cm^−2^ [42]. Zhang et al. [43] synthesized an efficient and long-term-durable heterostructured core@shell Ni_1−2x_Mo_x_Se@NH_4_NiPO_4_·6H_2_O–MoO_x_/NF electrode via electrodeposition approach. As a result, the developed electrode provided a fast mass transfer process during the water-splitting process and exhibited an excellent EC performance overpotential of 31 and 205 mV (after ohmic-drop correction) for the HER and OER, at current density of 10 mA cm^−2^, respectively. An amorphous-natured binary transition metal hydroxide (TMOH) electrocatalyst was synthesized via a simple precipitating metal nitrate deposition (PMND) technique. The optimized NiFe metal hydroxide electrocatalyst exhibited a water-oxidation current density value of 100 mA cm^−2^ and an overvoltage of 280 mV in alkaline conditions. From a tandem solar cell (P-V) study, the estimated power conversion efficiency was 13.71% [44]. A reef-like ternary structure for sulfur- and boron-doped (S,B-(CoFeCr)OOH) OxH-based electrocatalysts was successfully synthesized via solution combustion followed by wet-chemistry doping. The developed OxH can be used for the dissociation of saline water under neutral conditions. Furthermore, the achieved overpotential was 172 mV at 10 mA cm^−2^, and the Tafel slope was 45.3 mV dec^−1^ [45]. Zhang et al. [46] constructed an efficient and low-cost hierarchical PrBa_0.5_Sr_0.5_Co_2_O_5+δ_ (PBSC)@FeOOH electrocatalyst via both electro-spinning and atomic layer deposition (ALD) methods (Figure 1a). Furthermore, rough-surface PBSC nanofiber was uniformly deposited with FeOOH particles (Figure 1b). The optimized PBSC@FeOOH nanoflake electrocatalyst exhibited excellent EC performance in terms of both HER and OER activities. The estimated current density was 10 mA cm^−2^ at a favorable potential of 390 mV for OER and 280 mV for HER, as shown in Figure 1c. This much-improved performance was most likely due to the hierarchical nanostructure, high charge-transfer capability, and the strong electronic contacts between FeOOH and PBSC.

**Figure 2:**
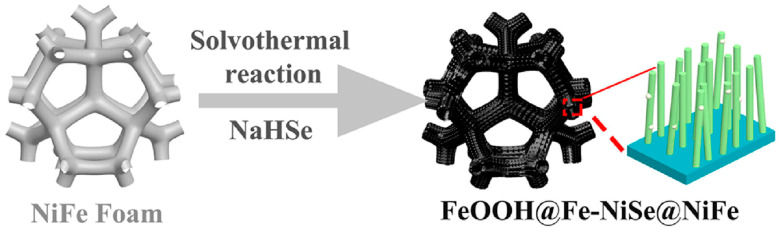
Schematic illustration of the preparation of FeOOH@Fe-NiSe@NiFe. Copyright 2022 by the American Chemical Society [49].

FeCo/CoFeP_x_O_y_(OH)_z_ electrodes were fabricated via an economic as well as a facile technique; the electrodeposited bifunctional-natured matrix displayed intrinsic catalytic activity for overall water-splitting process in alkaline solutions [47]. Kou et al. [48] purposely designed a cobalt-based molybdenum carbide (Co-Mo_2_C) precatalyst from a MoO_4_^2^—cations-substituted Co-based zeolite framework. Interestingly, the Mo-enriched CoOOH surface could promote electron transfer from Mo to Co sites through oxygen bridging, which significantly enabled water oxidation. A one-pot solvothermal method was developed for the fabrication of a hierarchical structure of FeOOH-decorated Fe-doped nickel-selenide on NiFe alloy foam (FeOOH@Fe-NiSe@NiFe) electrocatalyst (Figure 2). The as-fabricated aerophobic hierarchical heterostructured nature of the FeOOH@Fe-NiSe@NiFe electrocatalyst was used for the production of hydrogen during the electrolysis of water. An overpotential of 224 mV at 10 mA cm^−2^ was achieved with a small Tafel slope of 52.67 mV dec^−1^ and excellent stability [49].

**Figure 3:**
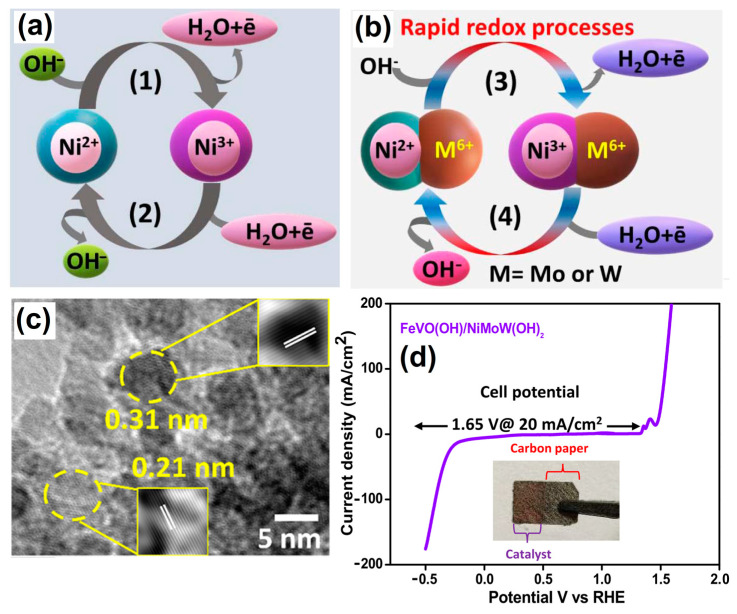
Schematic illustration of the proposed reaction mechanism of (**a**) Ni(OH)_2_ and (**b**) codoping of Mo^6+^ and W^6+^ in Ni(OH)_2_. (**c**) HRTEM image of FeVO(OH)/NiMoW(OH)_2_ and (**d**) LSV water-splitting curve of FeVO(OH)/NiMoW(OH)_2_@1.65 V vs. RHE, with the inset digital image showing the prepared electrocatalyst. Copyright 2023 by the American Chemical Society [50].

Multimetallic-natured metal oxides and hydroxides exhibit unique electronic structures and show good OER activities. For instance, binder-free heterostructures of FeVO(OH)/Ni_0.86_Mo_0.07_W_0.07_(OH)_2_ were decorated onto a commercial carbon paper (CP) substrate via a HT method. The water-splitting schematic diagram for the process under alkaline conditions is shown in Figure 3a,b. Here, the presence of higher metal oxidation states (Ni^3+^, Mo^6+^ and W^6+^) favoring the simultaneous reduction process of Ni^3+^/Ni^2+^ and the oxidation process of Ni^2+^/Ni^3+^ for the overall water-splitting mechanism is clearly shown. Thus, rationally designed hybrid-morphology-based nanoarray matrices have significantly enhanced OER and HER activities and could be a promising material for large-scale energy production through water splitting (Figure 3c,d) [50].

## 3. Transition Metal OxH Based Electrodes for OER

**Figure 4:**
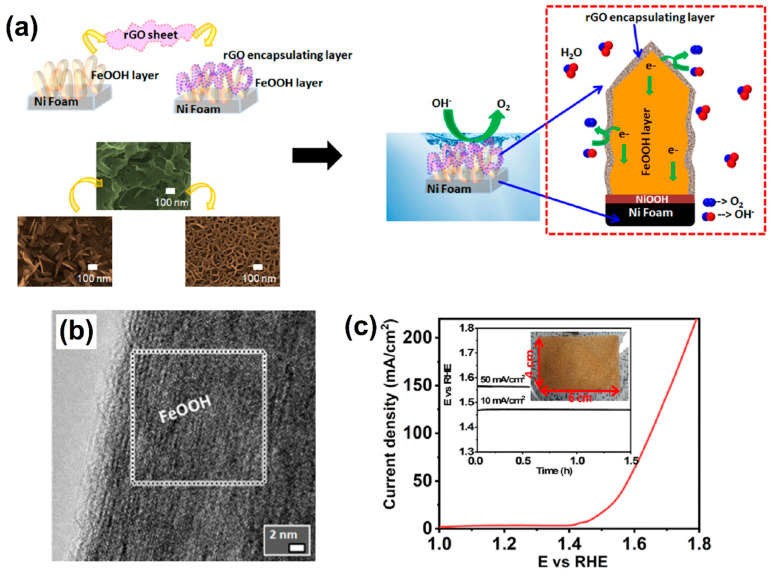
(**a**) Construction of β-like FeOOH nanosword on Ni foam and its further transformation into a 3D porous structure after rGO encapsulation to provide OER activities, (**b**) Fourier transform image of the selected area of β-like FeOOH, and (**c**) LSV curve of the scaled-up β-like FeOOH sample of 4 × 6 cm area on Ni foam. The inset photograph image shows the stability curve of the sample. Copyright 2021 by the American Chemical Society [65].

Recently, with the rapid development of sustainable and green energies, OER has become one of the important EC processes for the successive development of renewable energy storage technologies such as fuel cells, batteries, and water splitting [51]. Different kinds of electrocatalysts have been proposed for the study of OER under different pH conditions from acid to alkaline [52,53,54]. Among these, bimetallic-based OxH electrocatalysts have been proven to be a highly active catalyst that show outstanding EC properties during the OER under alkaline conditions [55]. The unique structure of amorphous metal OxH deposited on CeO_2_ and nickel foam (AMO@CeO_2_/NF) electrocatalyst is often considered the benchmark for OER. It exhibits a superior electrocatalytic activity and good cyclic stability in alkaline conditions. Additionally, AMO@CeO_2_/NF showed the best OER performance with a favorable potential of 261 mV at 10 mA cm^−2^ [56]. The layered structure of bimetallic-natured FeCoO_x_H was synthesized via a polyol method. The developed Fe_0.25_Co_0.75_OOH electrode showed the lowest overpotential for OER due to the electrostatic attraction between oxyhydroxide as well as its electrocatalytic activity with the metallic core components [57]. Iron-doped NiOOH (β-Fe/NiOOH) electrodes are the most efficient catalyst for alkaline OERs. Specifically, a β-Fe/NiOOH catalyst containing active Ni-based compound showed significantly enhanced OER activities with a favorable potential of 210 mV and a Faradic efficiency of 94.5% [58]. Huang et al. [59] found that the amorphous nature of cobalt–iron OxH nanosheet electrocatalyst can improve their OER activities under alkaline conditions. A notable overpotential of 208 mV at 10 mA cm^−2^ was achieved. A 3D-natured dendric-morphology-based Ni@Ni(Fe)OOH was deposited on nickel foam via ultra-ion exchange as well as electrodeposition methods. As a result, the dendric core–shell structure had more active sites to achieve a fast electron transfer process. The optimized Ni@Ni(Fe)OOH electrocatalyst significantly enhanced the OER and showed excellent stability in alkaline solution [60]. Hu et al. [61] used a novel EC technique: EC-deposited thin-film-natured, transition metal (oxy) hydroxides on nickel foam. They exclusively observed a four-electron transfer process during the OER. Generally, the iron substitution process can promote the peroxidation process to facilitate the formation of OxH-based active species. The process promotes the sluggish oxygen evolution reaction in alkaline conditions, exhibiting a low overpotential of 270 mV at 10 mA cm^−2^ with a small Tafel slope of 39 mV dec^−1^ [62]. An Fe-incorporated OxH-based electrocatalyst showed enhanced OER activities; systematic EC studies revealed a notable low overpotential of 283 mV at 10 mV cm^−2^ along with good intrinsic activities [63] for thin chromium-substituted α-cobalt OxHs. Moreover, the optimized α-Co_3_CrOOH electrocatalyst demonstrated a beneficial charge transfer resistance and acted as the potential used in the oxygen evolution reaction [64]. Deshpande et al. [65] used a simple and self-supported method to design a β-like FeOOH nanosword-like structure encapsulated on reduced graphene oxide (rGO) followed by nickel foam (NF) (Figure 4a). Figure 4b shows the highly crystalline-natured nanosword morphology of β-like FeOOH, which helped to enhance OER activities. Furthermore, the linear sweep voltammetry (LSV) technique helped with understanding the durability of the β-like FeOOH/rGO/NF electrocatalyst (Figure 4c).

**Figure 5:**
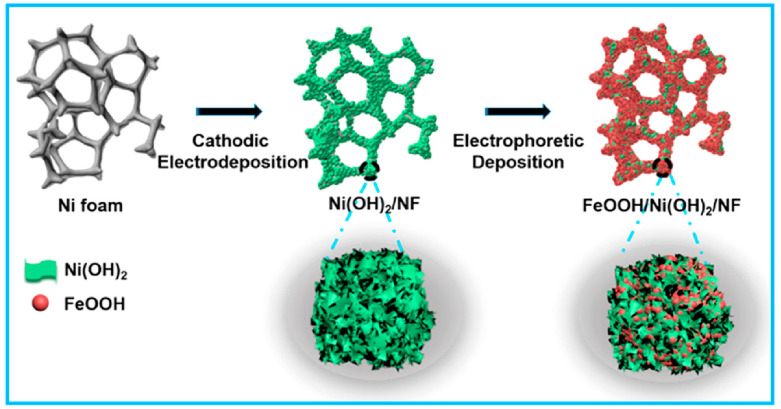
Schematic diagram of the fabrication of FeOOH/Ni(OH)_2_ on Ni foam through cathodic electrodeposition of Ni(OH)_2_. Copyright 2019 by the American Chemical Society [66].

Regarding synthesis strategies, the electrodeposition technique has been frequently used for the construction of β-Ni(OH)_2_ nanosheets, followed by α-FeOOH on nickel foam (Figure 5). Specially, electrostatic interaction occurs between Fe and Ni to enhance their OER process in alkaline medium. The constructed FeOOH/Ni(OH)_2_/NF hybrid electrocatalyst displayed excellent electrocatalytic activity toward OER activities; the required low overpotential was 207 mV at 40 mA cm^−2^ [66].

**Figure 6:**
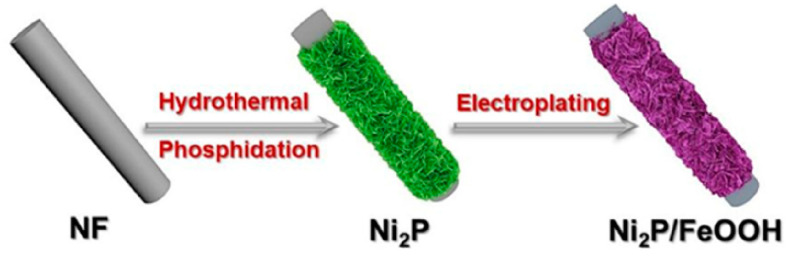
Scheme of the preparation of Ni_2_P/FeOOH. Copyright 2021 by the American Chemical Society [67].

Zhang et al. [67] used an important strategy for the successful construction of a heterojunction-natured Ni_2_P/FeOOH electrocatalyst (Figure 6). The Ni_2_P/FeOOH electrocatalyst exhibited larger area surface, which provided more active sites to accelerate the mass transfer process during the OER with an ultralow overpotential of 246 mV at 100 mA cm^−2^.

## 4. Transition Metal OxH Based Materials for HER

Recently, highly efficient and eco-friendly-natured hydrogen protection has become one of the most important energy technologies to produce affordable and clean energy. So far, the significant development of OxH-based electrode materials had led to the production of heterostructured active electrocatalysts for the study of the hydrogen evolution reaction (HER) during electrolysis in the water-splitting process under alkaline conditions. One of the classic examples of two-electron transfer reaction processes as follows [68]:

(6) $$2H^{+}+2e^{-}\to H_{2}$$

In general, HER and OER are the two important half-reactions in water electrolysis for the production of hydrogen [69]. A novel, stable, and efficient three-dimensional (3D)-based hierarchical structured γ-iron OxH-supported Ni_3_S_2_ was produced on nickel foam. This γ-FeOOH/Ni_3_S_2_/NF electrocatalyst exhibited good electrocatalytic activity toward both HER and OER activities. It displayed a low overpotential of 92 mV at 10 mA cm^−2^ for HER and 279 mV at 50 mA cm^−2^ for OER [70]. With the rationally developed Ni-foam-based porous-nickel-supported iron OxH (FeOOH/Ni/NF) electrocatalyst, the heterogeneous interaction between Ni and FeOOH could promote faster electron transfer reactions and a larger number of Fe and oxygen vacancies to enhance their HER and OER activities [71]. Controlling the electrodeposition technique is an important strategy for the fabrication of heterogeneous-natured crystalline (cNiFe) as well as amorphous-based bimetallic (aNiFe) OxHs on NiMo substrates. Fabricated bifunctional-natured NM@cNF/aNFO showed excellent electrocatalytic performance for the HER, OER, and overall water-splitting process [72].

Similarly, an in situ vertical growth technique was used for the fabrication of Ni(OH)_2_ nanosheets on NF, which could be further modified with bimetallic-natured double-layered hydroxides (NiFe-LDHs) via a two-step HT route. The as-constructed bifunctional NF-Ni(OH)_2_-NiFe-LDHs electrocatalysts achieved a low overpotential of 116 mV at 10 mA cm^−2^ for the hydrogen evolution reaction [73]. Lu et al. [74] used a facile and controlled method for surface reconstruction with Co^2+^- or Co^3+^-rich (oxy) hydroxide on ZnCo phosphate. Moreover, the developed reconstructed electrocatalyst tended to continuously increase HER performance under alkaline electrolytic conditions. Here, density functional theory (DFT) was used to study HER performance; the developed electrocatalyst can be applied in large-scale industrial wastewater decomposition. Defect-rich active-sites-based Mn-doped CoP (Mn-CoP)-nanowire-supported CoMn hydroxide nanowire on carbon red (CR) was fabricated via a facile HT and phosphorization process. As a result, the Mn-CoP nanowire@MnCoOOH composite showed good HER and OER performance [75].

**Figure 7:**
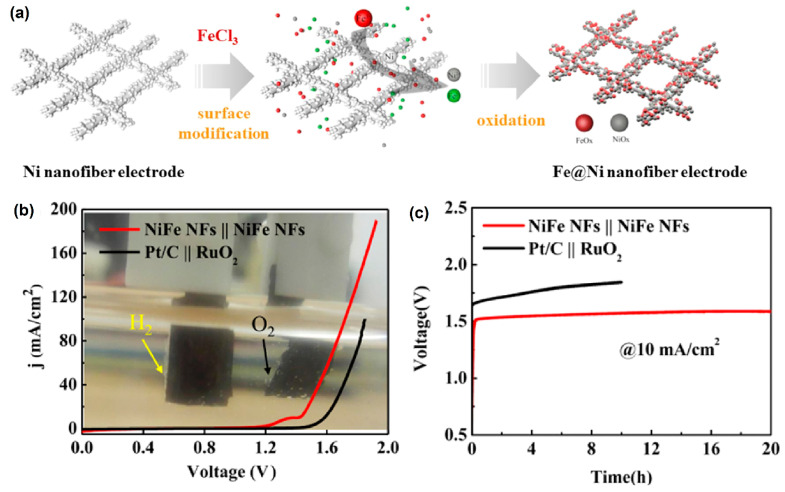
(**a**) Schematic diagram of the surface modification process of Fe@Ni nanofiber electrode; (**b**) EC performance of Fe@Ni nanofiber electrode, and Pt/C||RuO_2_ couples the polarization study for overall water-splitting reaction; and (**c**) electrode stability test under a current density of 10 mA cm^−2^. Copyright 2019 by the American Chemical Society [77].

Kundu et al. [76] reported an important analysis of the three different stoichiometrically ranges of nickel selenide compounds: Ni_0.85_Se, NiSe_4_, and NiSe_2_. Interestingly, the formation NiOOH could control the kinetic current and influence both the HER and OER performance of nickel selenide. Tao et al. [77] successfully developed a three-dimensional (3D) porous-network-based Fe@Ni nanofiber electrode via a chemical reduction method (Figure 7a), where abundant active sites of Ni/Fe (oxy)hydroxide were formed on the electrode surface. To examine the surface morphology, SEM studies revealed that the diameter of Fe@Ni nanofibers was about 200 nm. The as-prepared active Fe@Ni nanofiber electrocatalyst needed a low overpotential of 55 mV and 230 mV to achieve a current density of 10 mA cm^−2^ in HER and OER, respectively (Figure 7b).

**Figure 8:**
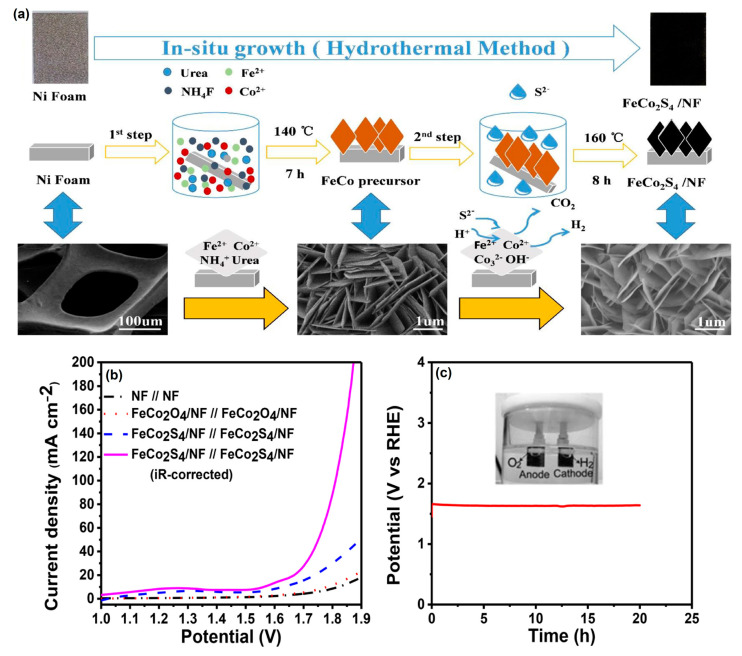
(**a**) Schematic illustration of the formation of FeCo_2_S_4_ nanosheet arrays on Ni foam and their corresponding morphology, (**b**) two-electrode OER polarization curves of FeCo_2_S_4_/NF//FeCo_2_S_4_/NF,FeCo_2_O_4_/NF//FeCo_2_O_4_/NF and NF//NF at a scan rate 2 mV s^−1^, and (**c**) two-electrode cell durability of FeCo_2_S_4_/NF//FeCo_2_S_4_/NF electrode image with gas evolution (inset) in 1 M KOH. Copyright 2018 by the American Chemical Society [78].

Hu et al. [78] successfully prepared bifunctional-natured FeCo_2_S_4_ nanosheet arrays on 3D nickel foam (NF) via a facile HT method (Figure 8a). From the X-ray photoelectron spectroscopy (XPS) analysis, real active species of the Co(Fe) oxyhydroxide layer formed on the electrocatalytic surface. Moreover, the as-prepared FeCo_2_S_4_/NF electrode showed excellent electrocatalytic activity toward the HER with long-term stability for water splitting under alkaline conditions (Figure 8b).

## 5. Mixed Transition Metal OxHs for the OER and HER 

Future energy production methods such as EC water splitting, which produces hydrogen using renewable energy, are exciting. For the OER and HER processes involved in water splitting, dependable catalysts are needed for the reactions to proceed at a decent pace with fewer overpotentials [79,80]. Precious metal oxides such as IrO_2_ and RuO_2_ are now the most advanced electrocatalysts for the OER under both alkaline and acidic conditions. However, their higher anodic potentials make them unstable in electrolytes and lead them to dissolve in solution, where they then oxidize into IrO_3_ and RuO_4_, respectively [81]. Furthermore, due to their high cost and scarcity, precious metal oxides have limited large-scale commercial applications. Applications of earth-abundant transition metal oxides (Ni, Fe, Co, Mn, etc.) for EC energy storage and conversion have recently attracted a lot of interest [82]. Nickel, a first-row transition metal, and its alloys, in particular, have shown incredible potential as electrodes for batteries and supercapacitors in alkaline media [83]. Up till now, mixed metal oxides, OxHs, and other functional additions have been the most efficient electrocatalysts for alkaline OERs or HERs due to their advantageous characteristics, including enhanced mass transfer, improved conductivity, improved structure/performance stability, and mixed metal oxide/OxH synergistic action. As an example, Babar et al. [34] developed 2D cobalt–iron hydroxide nanosheets on nickel foam substrates as electrocatalysts for water splitting and the wet chemical deposition of FeOOH NPs (CoFe-OH@FeOOH). With its huge surface area and numerous active sites, the nanocomposite enabled rapid EC water-splitting kinetics. It is noteworthy that the electrode’s low overpotential value for OER was 200 mV at 50 mA cm^−2^. When used as both the anode and the cathode for complete water splitting, it gave a low cell voltage of 1.56 V to produce a 10 mA cm^−2^ current density. However, it was found that the CoFe-OH/FeOOH interface had synergistic activity, which markedly improved conductivity and mass transfer. This simple technique offers a different perspective on how to create appropriate electrodes for use in energy conversion applications.

**Figure 9:**
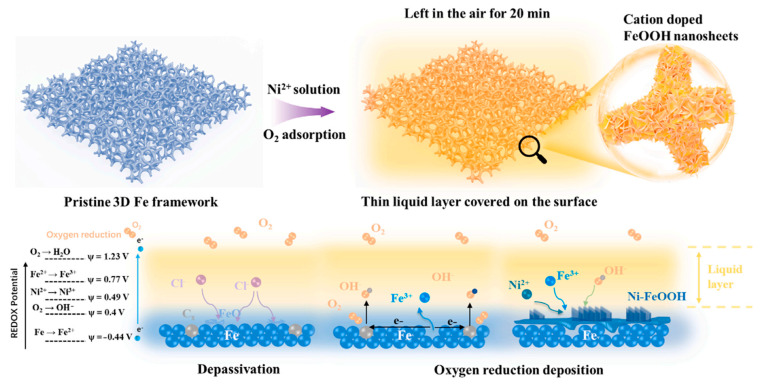
Illustration of Ni-FeOOH nanosheet synthesis via oxygen-reduction deposition method. Copyright 2023 by the Elsevier [84].

Yan et al. [84] synthesized Ni-incorporated FeOOH nanosheets in alkaline environments using a self-developed oxygen-reduction deposition process that resulted in exceptional OER activity with a small Tafel slope of 33 mV dec^−1^ (overpotential of 239 mV at 10 mA cm^−2^). This technology has the distinct features of a short synthetic period (20 min) and ultralow synthetic costs (no need for high temperature/pressure or noble materials), making it a promising platform for investigating low-cost and high-efficiency FeOOH-based nanoelectrocatalysts. The production of Ni-FeOOH nanosheets is depicted in Figure 9. The force driving synthesis in this approach is the natural redox potential difference between dissolved O_2_ and the Fe substrate. The presence of Ni heteroatoms (7 at.%) on the FeOOH surface raised the Fe average valence to above +3, lowered the Fermi level, and increased the amount of electron acceptors. As a result, the energy barrier of the rate-determining step for both the Ni and Fe sites was lowered compared with that of pure FeOOH, resulting in OER activity in alkaline media.

**Figure 10:**
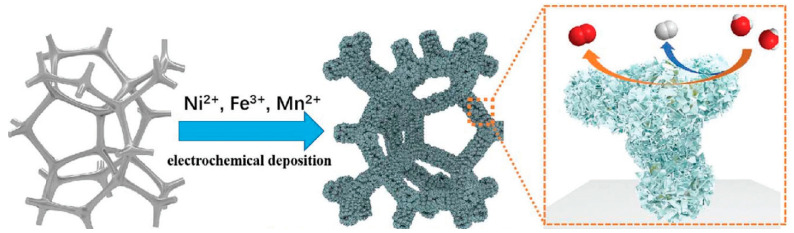
Schematic illustration of the fabricating process of NiFeMn-AOs electrode and its catalytic function for both OER and HER. Copyright 2021 by the Royal Society of Chemistry [85].

Similarly, Ge et al. [85] investigated the development of a low-cost and high-performance bifunctional NiFeMn alloy@NiFeMnOxH electrocatalyst using a one-step chronoamperometry electrodeposition process (Figure 10). Doping with Mn effectively engineered a multilevel dendritic NiFeMn alloy and an OxH layer with this approach, greatly lowering the energy barrier for the OER and HER, as demonstrated by DFT simulations. Thus, at a current density of 10 mA cm^−2^, the produced catalyst exhibited good electrocatalytic performance for the OER (219 mV) and HER (19 mV). This catalyst displayed exceptional performance for overall water splitting, both experimentally and conceptually, opening up new avenues for future industrialization.

**Figure 11:**
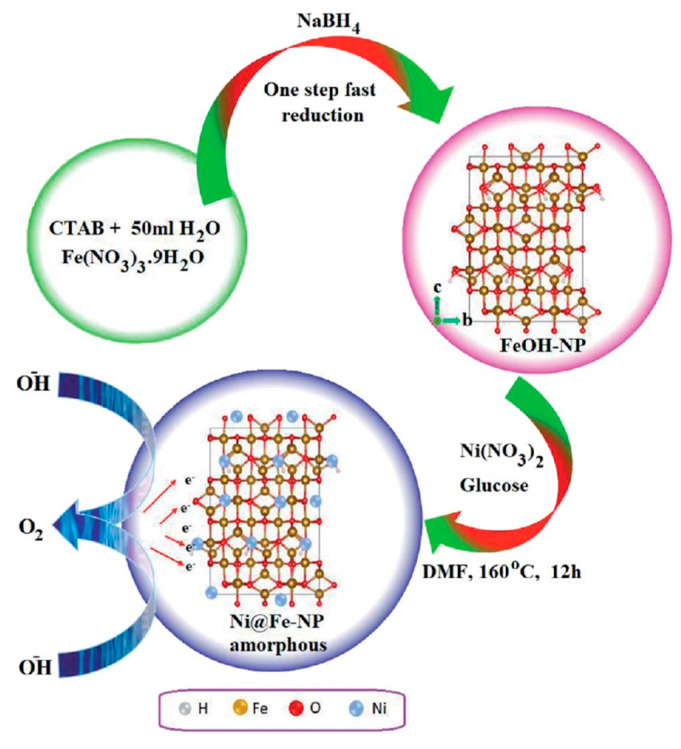
Schematic route for amorphous Ni@Fe-NP. Copyright 2020 by the Royal Chemical Society [87].

Gao et al. [86] discovered a new approach to synthesize nickel/nickel (oxy)hydroxide hybrid films with improved catalytic performance for total water splitting by manipulating oxygen deficits at the catalyst’s surface. The initially deposited Ni films from an ethylene-based deep eutectic solvent (DES) undergo structural rearrangement with a phase shift in the oxidation state from Ni(II) to Ni(III) near the surface under OER conditions. The shift is accompanied by a rise in oxygen deficits, and the addition of nitrate ions induces a pronounced faulty precursor, providing structural disordering and enhancing the intrinsic activity of the catalyst, which greatly improves the water-splitting performance. Mathi et al. [87] described solvothermal Ni@Fe-NP electrocatalysts that are highly efficient, ultradurable, and earth-abundant. Figure 11 depicts the development of amorphous Ni@Fe-NP. The newly designed oxygen electrode exhibits long-term stability and high electrocatalytic activity in water oxidation under alkaline conditions, requiring an overpotential of just 211 mV at a current density of 10 mA cm^−2^. Surprisingly, in alkaline solution, the as-prepared amorphous Ni@Fe-NP rippled nanostructured electrode was the most effective oxygen evolution electrode. As a result, this research opens up fascinating new possibilities for creating self-supported electrode materials for water-splitting and other applications.

Poudel and Kim produced ternary Zn-Mg-Al-based double-hydroxide nanosheets and hematite–iron oxide on hollow porous carbon nanofibers via an electrospinning process, which resulted in a three-dimensional hierarchical nanocatalyst. They found that the percentage of Zn determines the structural, morphological, and electrochemical properties; the 1D-hollow/2D-mesoporous/3D-bimetallic nanocomposite expressed a capacitance of 3437 F cm^−1^ at 1 mA cm^−2^. One-dimensional hierarchical CoMoP nanosheets were homogeneously wrapped on Ni_3_S_2_ nanowires directly on nickel foam by Yoo et al. and used for the OER and HER. They found that the proposed catalyst produces hydrogen intermediates that significantly reduce the overpotential of 6.8 mV for alkaline HERs. Additionally, this catalyst showed excellent stability up to 30 cycles in an alkaline medium [1,2]. Wu et al. conducted a detailed study on Ni-Fe selenide-based active structures for the OER. Both the Ni and Fe nanometallic counterparts were sluggish in terms of OER activity, and this was improved by combining these metals, which provided excellent catalytic activity. Ni is highly active toward the OER, and Fe synergizes with Ni to remove the poisonous counterparts, which additionally helps to improve the active sites of Ni. Similarly, Wang et al. found that NiFe-borides/borates were the best electrocatalysts for the OER in alkaline media. Through DFT calculations, they found that the in situ formed new phase of NiB_4_O_7_ could function as the active site for active oxy-species (O*, *OH, *OOH) generation in an electrolytic medium. The proposed catalyst exhibited an oxygen evolution overpotential of 167 mV at 10 mA cm^−2^ in 1 M KOH. Additionally, the proposed electrocatalyst is considered promising for the OER [88,89,90,91].

## 6. Rare-Earth Metal Oxide/OxH for OER and HER

Rare-earth metal-doped metal oxides, in particular, display excellent activities, which improve a material’s conductivity and surface area, as well as the diffusion routes. Many rare-earth metals have been utilized as doping agents, including gadolinium (Gd), neodymium (Nd), dysprosium (Dy), and samarium (Sm) [92]. These metals/metal oxides significantly boost ionic conductivity, carrier transportability, and catalytic activity. The discovery of abundant rare-earth electrocatalysts for OERs and HERs in strong acids in recent years has created a substantial challenge in the development of high-efficiency, long-lasting, and cost-effective electrolyzers and fuel cells [93]. To achieve the benchmark current density (10 mA cm^−2^), rare-earth Dy-doped cupric oxide nanoparticles (Cu_1−x_Dy_x_O) electrocatalysts demonstrated an outstanding OER at 1.55 V versus RHE and HER at 0.036 V vs. RHE in an aqueous 1.0 M KOH electrolyte [94]. 

**Figure 12:**
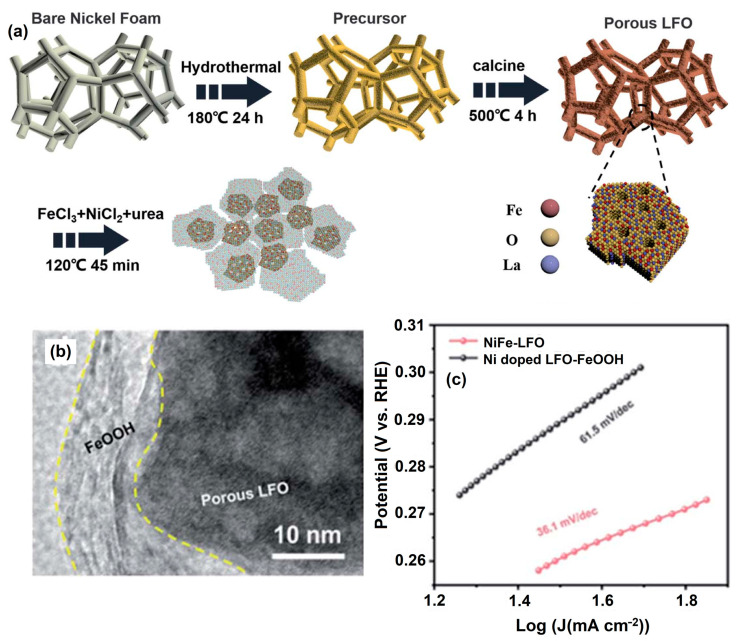
Schematic representation of synthesis route of NiFe–LFO. (**a**) Synthesis process, (**b**) TEM image of NiFe–LFO and (**c**) power density response. Copyright 2021 by the Royal Chemical Society [95].

NiFe-LFO was synthesized via solvothermal method and calcined at different temperatures. By immersing porous LFO in a solution of Ni^2+^, Fe^3+^, and urea, NiFe-LFO samples with varying Ni contents and Ni^2+^/Fe^3+^ molar ratios were prepared. The best -performing sample had a Ni^2+^/Fe^3+^ ratio of 49 and a Ni content of around 15% (Figure 12a). TEM images showed amorphous ultrathin nanosheets in NiFe-LFO (Figure 12b), while immersion in Ni^2+^ solution did not produce similar nanosheets, confirming the composition as FeOOH. NiFe-LFO exhibited a significantly lower overpotential cthanLNO-FeOOH and Ni-doped LFO-FeOOH (Figure 12c), highlighting the beneficial effect of surface Ni doping on catalyst performance.

DFT calculations show that the strong electronic interactions at the composite’s interface play a critical role in improving catalytic activity. Furthermore, Ni^2+^ doping efficiently changes the electronic structure of LFO and improves electron transport at the interface of LFO and FeOOH, resulting in an elevated d-band center of FeOOH and strong OER activity. This work laid the groundwork for the development of improved OER electrocatalysts for sustainable energy devices [95].

Niss et al. [96] explored two alternative catalytic designs in depth in order to understand the diverse spatial arrangements that determine the characteristics of the OER. The two designs were Sm_2_O_3_-loaded ZnO on the surface (Sm-Zn-L) and Sm_2_O_3_-embedded ZnO (Sm-Zn-E). In comparison with the Sm-Zn-L catalysts, the Sm-Zn-E catalysts had a reduced overpotential (419 mV for 10 mA cm^−2^), Tafel slope (89 mV dec^−1^), and good stability up to 40 h and 1000 cycles. This could be explained by the entrenched arrangements benefitting the OER. The incorporation of minute Sm_2_O_3_ clusters into ZnO enhanced the surface area, reduced the amount of surface defects, and increased the efficiency with which the electronic assemblies of the surface-active sites were optimized. Based on these findings, Sm doping provides a feasible paradigm for rearranging catalysts to improve their spatial performance [97].

## 7. Polymer-Based Metal OxHs for OER and HER 

An exceptional technology that is in demand for producing clean energy without the release of greenhouse gases is the conductive-polymers-based electrolysis of water (OER/ORR) [98]. Conducting polymers were combined with metal/metal OxHs, which are suitable for water splitting reactions and accelerate the rapid transferring of electrons [99,100]. Metal–air secondary batteries (MABs) are interesting options for next-generation energy storage systems due to their high theoretical energy density of over 1000 W h kg^−1^. The OER and ORR operate an alkaline metal–air battery’s air-positive electrode as follows [101]:

(7) $$O_{2}+2H_{2}O+4e^{-}⇋4{OH}^{-}\;E^{0}=1.23\;V\;vs.\;RHE$$

The unique interaction between the AAO template and the polycyclic aromatic intermediate chemicals generated during PVC heat degradation creates the graphene structure. Polarization at +40 mA cm^−2^ in 4 mol dm^−3^ KOH was used to test carbon/CFCO electrodes’ OER endurance. DB/CFCO, DB2400/CFCO, and pCNF1500/CFCO’s electrocatalytic activity toward OER was dramatically reduced, with a current density at 1.7 V vs. RHE dropping to 53%, 54%, and 67% of the original value, respectively [102]. Perovskite oxide/carbon electrodes’ OER current decreased due to carbon corrosion. The MWCNT/CFCO electrode’s current density dropped to 87% at 1.7 V vs. RHE due to OER deactivation, but this sample had the highest graphitization degree and therefore better oxidation resistance [103].

A nickel oxyhydroxide–chitosan nanocomposite electrocatalyst with high electrocatalytic activity and stability for glucose oxidation (Gox), ORR, OER, high sensitivity, and selectivity for glucose electro-sensing was investigated. This was the first study to examine the EC characteristics of the as-prepared nanocomposite for fuel cell applications, which was previously employed in the adsorption process to remove heavy metals such as Ni(II), Fe(III), and Cu(II) ions from aqueous solutions using different types of chitosan biosorbents [104]. Expectedly, the calcined (curve d) and uncalcined (curve) nano-NiOOH/GC electrodes had stronger OER activity than both bare GC and CS/GC electrodes, demonstrating NiOOH’s crucial function in catalyzing the OER. After annealing at 250 °C, nano-CS-NiOOH/GC electrodes displayed a rapid rise in the OER current and a large negative shift in the OER onset potential, suggesting increased electrocatalytic activity for the OER [105]. The catalysts’ structure and electrocatalytic activity depended on the nano-CS-NiOOH calcination temperature. The nano-CS-NiOOH/GC electrode’s electrocatalytic activity toward ORR, OER, and Gox rose with calcination temperature (>280 °C) due to CS matrix degradation [106].

To improve EC water-splitting efficiency, high-performance and low-cost OER electrocatalysts are needed. Here, a porous and self-supported FeNi-alloy fiber paper was effectively manufactured to exhibit exceptional OER performance with an ultralow overpotential and amazing stability. EC water splitting is a sustainable and environmentally benign way to manufacture hydrogen for energy storage utilizing power from solar, wind, and other renewable sources [107]. The HER and OER occur at the cathode and anode electrodes, respectively, during water splitting. The HER utilizes two-electron transfer processes, while the OER needs a sophisticated multistep proton-coupled electron transfer process (4OH^−^ → O_2_^2−^ + 2H_2_O + 4e^−^), which slows reaction kinetics and reduces water-splitting efficiency. OER electrocatalysts must be highly efficient. The benchmark OER electrocatalysts are noble-metal-based oxides (IrO_2_, RuO_2_, etc.) [108,109].

For the oxygen evolution reaction (OER) process, the lattice-oxygen-mediated mechanism (LOM) on perovskite oxides was found to be kinetically superior to the more common adsorbate evolution mechanism (AEM). However, it remains difficult to realistically increase lattice oxygen involvement. A lead–selenium–cobaltate–fluoride–methylene (LSCFM) electrocatalyst was prepared for use in the OER. The larger concentration of the surface oxygen vacancies and faster oxygen ion diffusion coefficient in LSCFM are responsible for its increased OER catalytic activity, as confirmed by XPS, pH-dependent OER kinetics, and ionic diffusivity experiments [110].

**Figure 13:**
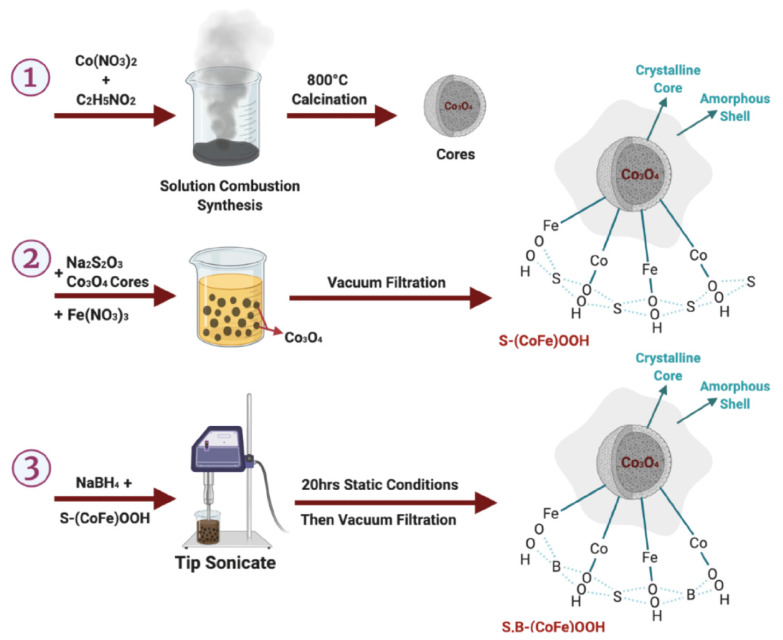
Synthesis strategy of S- and B co-doped CoFeOxHs. Copyright 2022 by the American Chemical Society [111].

To improve OER activity while keeping the ORR rate high, a network of nanoparticle-sized La_0.8_Sr_0.2_MnO_3_ (LSM)-based perovskite catalysts was synthesized using a polymer-assisted chemical solution (PACS) approach. Due to its relevance to numerous applied technologies and industries, the OER and ORR have received extensive research attention. Chemical energy storage heavily relies on water splitting via oxygen evolution. Many essential and convenient energy conversion and storage technologies, such as water electrolysis and rechargeable metal–air batteries, rely on the OER and ORR as fundamental phenomena [111]. A S and B co-doped CoFeOxH with apparent CER suppression properties was easily and quickly made using a solution combustion synthesis method (Figure 13; 1 to 3). With a low Tafel slope of 46.7 mV dec^−1^, the as-prepared S,B-(CoFe)OOH-H attained a low overpotential of 161 and 278 mV with current densities of 10 and 1000 mA cm^−2^, respectively.

Additionally, systematic electrolyte testing between pH values of 14 and 7 produced promising results, bringing direct seawater electrolysis under nearly neutral pH conditions closer to reality [112]. Perovskite nanostructure, nickel iron sulfides on nickel foam (NiFeS/NF), Co-Mo-B nanocomposition, cobalt disulfide/graphite, and NiFeO_x_ nanoparticles are examples of new classes of bi-functional catalysts for both the HER and OER that have emerged in recent years. While RuO_2_ is well known as an OER catalyst, its use in HER is far more limited. Improvements in electrocatalytic performance are required for HER catalysts to overcome issues regarding reactivity, poor electron transport, low surface area, and instability under operating circumstances. In the oxygen evolution reaction, the Tafel slope of RuO_2_ NR is 92.6 mV dec^−1^. As a result, this catalyst has emerged as a leading alternative to conventional oxide and sulfide nanoparticles for use in HERs and OERs. Many useful and convenient methods of converting and storing energy, such as the electrolysis of water and rechargeable metal–air batteries, rely on the OER. The timely innovation and discovery of effective water oxidation electrocatalysts are required to advance sustainable energy systems. Together with a state-of-the-art brown millerite CFCO electrocatalyst for OER/ORR in a 4 mol dm^3^ KOH electrolyte, carbon nanomaterials with different carbon plane orientations were investigated as conductive substrates. The long-term durability of the carbon nanostructure in extremely alkaline electrolytes was shown to depend on the proper arrangement of exposed carbon planes with a rather high degree of graphitization. In metal–air batteries with alkaline aqueous electrolytes, highly graphitized platelet-type carbon nanofibers show promise as conductive carbon supports for long-lasting air electrodes. During the OER, surface Fe atoms are readily oxidized, and the surface of the FeNi alloy is leached away to expose more Ni atoms, which then become active OxH sites. A critical active material for the OER is metal OxH, and FeNi alloy can speed up electron transfer along reaction pathways. The improved electrocatalytic activity for the HER and OER at lower overpotentials of 0.104 V and 0.246 V, respectively, to deliver 10 mA cm^−2^ can be attributed to the FeCo-LDH catalyst’s strength, many defects, electron transport from Fe, and increased conductivity. The EC reconstruction of alloys, an alternative approach to designing extremely efficient electrocatalysts, will speed up the use of cheap, effective, and long-lasting electrocatalysts [113].

## 8. MXene-Supported Metal OxHs for OER and HER

Several etching and delamination techniques can be used to transform MAX phases into the 2D material family known as MXenes, which consists of transition metal carbides and nitrides [114,115]. The general formula for a MAX phase is M_n+1_AX_n_, where M represents an early transition metal, A represents an element from group 13 or 14, and X denotes either carbon or nitrogen. By removing the group 13/14 element from the MAX structure via etching in a fluoride ion-based solution, the carbide layers are terminated by OH, O, Cl, or F groups, which are referred to as “surface groups” or “edge sites”. ‘MXene’ is the name given to the final structure. It is hypothesized that combining pure metal oxides with MXenes as a catalyst layer for the OER would be beneficial because of MXenes’ superior conductivity, hydrophilicity, and tunability. Interestingly, MXene materials are promising for the HER, the inverse of the water-splitting reaction, via computational calculations and experimental approaches [116,117,118]. Hydrogen production relies on the discovery of plentiful, low-cost, and highly active catalysts for the HER and OER. Many researchers are interested in using nanolaminate ternary transition metal carbides (MAX phases) and their derived two-dimensional transition metal carbides (MXenes) as electrocatalysts. Four new MAX@MXene core–shell structures have been constructed, with Co/Ni-MAX phases serving as the core and MXenes serving as the shell. An overpotential of 239 mV and high stability during the HER with MXenes as the active sites were observed with the Ta_2_CoC@Ta_2_CT_x_ core–shell structure under alkaline electrolyte conditions. Maintaining a bulk crystalline structure and generating Co-based OxHs that were developed via surface reconstruction as active sites, the Ta_2_CoC@Ta_2_CT_x_ core–shell structure exhibited an overpotential of 373 mV and a modest Tafel plot (56 mV dec^−1^). This work presented a new approach to creating multifunctional electrocatalysts by taking into account the diverse chemical compositions and structures of MAX phases [119], and it set the way for the future development of MAX-phase-based materials for use in clean energy applications. 

**Figure 14:**
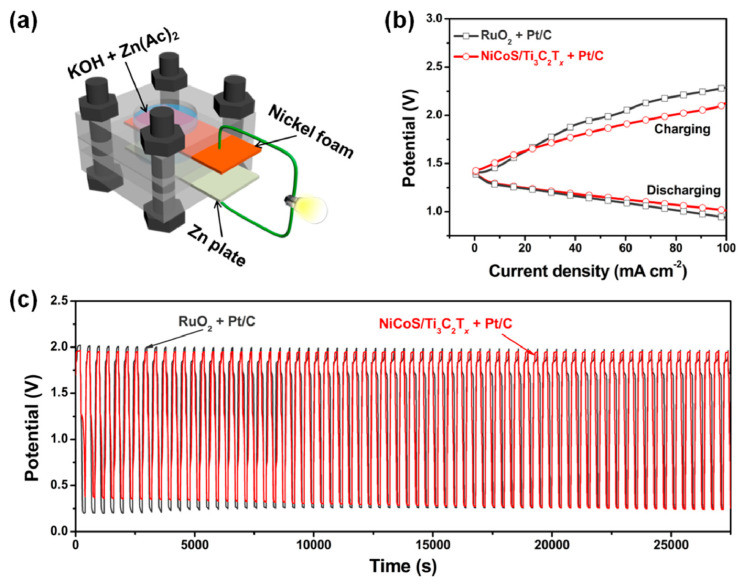
(**a**) Scheme of a homemade rechargeable Zn–air battery. (**b**) Charging and discharging polarization curves of rechargeable Zn–air batteries. (**c**) Cycling performance at a current density of 10 mA cm^−2^. Copyright 2018 by the American Chemical Society [120].

Using a metal–organic framework (MOF)-based strategy (Figure 14a), Zou et al. [120] developed a new hierarchical porous NiCo mixed-metal sulfide (denoted as NiCoS) on Ti_3_C_2_T_x_ MXene. The hybrid’s better activity toward the oxygen evolution process (OER) results from the combination of NiCoS and Ti_3_C_2_T_x_ sheets, each of which benefits from the other’s distinctive structure and strong interfacial interactions. The OER measurements revealed that the NiCoOOH on the surface is the intrinsic active species for the subsequent water oxidation, demonstrating how the hierarchical NiCoS in the hybrid is changed to a nickel/cobalt oxyhydroxide-NiCoS assembly (denoted as NiCoOOH-NiCoS). The hybrid material is then used in an air cathode for a rechargeable zinc–air battery, which displays long-term stability and low charging/discharging overpotential (Figure 14b,c). This work highlights the adaptability of MXenes in tuning the structure and electrocatalytic OER performance of MOF derivatives and sheds light on the structure–activity link for catalysts that do not require noble metals. Ni_x_Fe_y_ nanoalloys supported on highly electron-conductive 2D transition metal Mo_2_CT_x_ MXene were synthesized via a polyol-assisted solvothermal method. Several physicochemical methods are used to ascertain the materials’ structures, morphologies, and compositions. In addition, to facilitate the production of nanostructured metallic Ni_x_Fe_y_ nanoalloys on an MXene support, this material also enhances the OER by facilitating charge transfer at the interface [121].

An HT reaction followed by sulfurization was used to create composites of CoS_2_ nanowires attached to the electronegative surfaces of exfoliated MXene nanosheets. When compared with pure CoS_2_ and MXene, the CoS_2_@MXene composite produced a more efficient trifunctional electrocatalyst with a lower overpotential for ORR, OER, and HER in alkaline solutions. In order to build efficient noble-metal-free catalysts for energy conversion systems [122], the multidimensional fabrication of 1D CoS_2_ nanowires on a 2D MXene substrate has led to new insights. HT growth was followed by an in situ phosphorization strategy, as demonstrated by Li et al. [123], who prepared a NiFeCoP/MXene catalyst and demonstrated that MXene coupling and phosphorization could significantly induce the formation of high-valence TM active sites by altering the arrangement of electrons close to TM (Ni, Fe, and Co). Furthermore, as shown by the EC measurements, numerous OER active species NiOOH could be easily created, endowing NiFeCoP/MXene with a low overpotential of 240 mV at 10 mA.

## 9. Solid Supports for Metal OxHs for OER and HER

Metal oxides and their hydroxides offer exceptional electrocatalytic activity toward various energy conversion and/or storage systems. In metal-oxide-based modified electrode systems, metal/metal oxides have been introduced into aqueous electrolytic environments. Oxide/hydroxide formed on the electrode surface area, playing a significant and important role in electrocatalytic reactions [124]. In this section, some of the solid-supported metal OxHs and their catalytic activity toward the OER and HER are briefly discussed. 

Cheng et al. [125] designed Ba-, Sr-, and La-based perovskites-supported cobalt–iron-based bimetallic OxHs with amorphous carbon. In general, Fe enhances the OER in the cobalt OxH systems because the Fe is oxidized comparatively faster and stabilizes the cobalt cation in the lower oxidation coordinates. Additionally, they indicated that the electronic interaction between Co and Fe enhances the OER, which was achieved with a lower content of Fe in the BSCF perovskite. A three-dimensional NiFe-based OxHs-supported nickel foam was reported by Yuan et al., and this catalyst expressed exceptional OER activity of 10.0 mol L^−1^ with a KOH electrolyte (80 °C). Electron-donating functional groups (–NH_2_, –OH, and –OCH_3_) and electron- withdrawing functional groups (–COOH and –NO_2_) play a key role in this catalytic activity. Additionally, these functionalities help to enhance and/or modify the electrode–electrolytic interface to improve electrocatalytic activity. In situ EC Raman spectroscopy showed the active involvement of FeOOH and NiOOH, which produced excellent activity with a super-high catalytic current density of 500 mA cm^−2^ [126].

Recently, Lu et al. [127] synthesized NiFe OxH-anchored N-doped carbon aerogel, which showed excellent OER activity with a low electrode overpotential of 304 mV at10 mA cm^−2^. Additionally, DFT calculations revealed that the increase in the binding ability between the metal (Ni-Fe) OxH and carbon aerogel enabled the development of an active catalyst for energy storage and energy conversion systems. The slow electro-kinetics of the OER have been addressed via the effective synthesis of electrocatalysts. Along this line, Yu et al. developed NiFe OxH clusters anchored on carbon black. With its excellent nanostructure and increased surface area, this catalyst showed excellent OERs at a low overpotential of 320 mV. The theoretical calculations and experimental results showed that Ni-O-Fe structures offer exceptional catalytic activity in terms of oxygen electrochemistry [128]. Xu et al. [129] developed cobalt hydroxide nanoflowers-decorated α-NiMoO_4_ nanowires as functional electrocatalysts for water splitting reactions. They decorated the Co(OH)_2_/NiMoO_4_ nanowires on a carbon cloth via a simple HT process, which formed as one-dimensional nanoarchitectures, providing a lower overpotential toward the HER of 183 mV and the OER of 170 mV at a current density of 10 mA cm^−2^ in 1.0 M KOH electrolytes, showing good long-term stability. A 3D hierarchical nanoarchitecture, consisting of ultrathin porous nickel–iron oxides/hydroxide, was assembled to a metallic nickel–copper alloy, which exhibited excellent OER activity at an overpotential of 218 mV as well as the HER at 66 mV at 10 mA cm^−2^. Additionally, the prepared catalyst exhibited 293 and 506 mV at 10 and 50 mA cm^−2^, respectively, for a solar-panel-powered electrolyzer (1.5 V) [130]. 

Solar water splitting using tantalum OxHs with Sr and BO/(OH)_x_ was achieved by Lawley et al. [131]. They found that Sr in the catalyst enhances the surface reconstruction process, and BO/(OH)_x_ improves the outer-surface hydrophilicity of the prepared catalyst with the modified electrodes, which enhanced the overall water-splitting reaction. Additionally, these oxy-nitroxy interfaces provided enhanced light adsorption and showed excellent catalytic activity for the OER. Amorphous CoFe-based double-hydroxide-supported N-doped CNTs were developed via a facile, room-temperature method by Liu et al. for the OER [132]. The prepared double hydroxide provided a better catalyst for aqueous environments and formed OxHs; these OxHs helped to extract active oxygen species from the electrolytic solution. Additionally, the amorphous nature of the metal hydroxide possessed more defective surface area, which helped to boost the OER. A bifunctional and binder-free SnFe sulfide/oxyhydroxide heterostructured nanoarchitecture on nickel foam was developed via a facile solvothermal method by Zhan et al. [133]. The prepared electrocatalysts exhibited a lower overpotential for the HER of 324 mV and for the OER of 281 mV at 1000 mA cm^−2^. This was achieved via a sulfide/oxyhydroxide heterostructured interface with excellent electrocatalytic activity toward the active oxygen separation in the OER.

**Figure 15:**
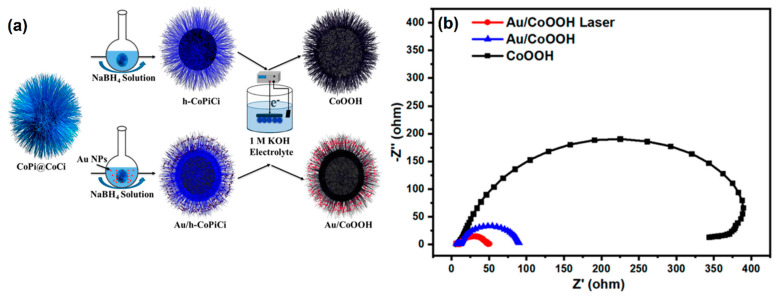
(**a**) The synthetic route of Au/CoOOH; (**b**) EC impedance plots of CoOOH, Au/CoOOH, and Au/CoOOH. Copyright 2020 by the American Chemical Society [135].

A tunable proton acceptor such, oxoacid (C_2_O_4_^2−^) on NiOOH, was developed, which achieved the effective irreversible reconstruction of Ni metal compounds (i.e., active surface) to improve the OER. Additionally, these acids could help to extract protons or hydroxide ions from the electrolytes, leading to an enhanced OER activity of 270 mV (10 mA cm^−2^) [134]. The authors stated that this strategy can be applied for other transition metals such as Co, Fe, etc. Feng et al. [135] synthesized an architecture of hierarchical hollow-shell-natured cobalt-metal-supported OxH (CoOOH) on gold nanoparticles (AuNPs) via a facile method (Figure 15a). The EC impedance spectroscopy (EIS) analysis of AuNPs/CoOOH also confirmed the photo-assisted OER activity, which achieved an incident photoelectron conversion efficiency of up to 5% (Figure 15b).

## 10. Conclusions

Owing to the ever-increasing energy demand and its associated climate change, researchers are continuously working toward the development of active and cost-effective energy storage and conversion systems. One of the important, cost-effective, alternative green technologies that is being developed for commercialization is water electrolysis. Various achievements have been made to improve the electrode active kinetics in the hydrogen evolution reaction, oxygen evolution, and water splitting through the design of catalysts. To improve the efficacy of the process, metal OxHs provide excellent and stable electrode systems for long-term usage. Additionally, transition metals, such as Ni, Fe, Co, Mn, etc., and their electrocatalysts are playing a central role in the electrocatalytic systems for the HER, OER, and water-splitting reaction. Earlier researchers found that the transition metal and its oxides and their sluggishness can be addressed by either adding various other metals and their OxHs to the systems, carbon-based supports, or other metal oxides. Supports co-catalysts, MXenes, polymers, etc., were reviewed, and their progress was briefly described in this paper. The following improvements are suggested to enhance the reaction: (i) Detailed mechanistic studies of redox reactions in water splitting are urgently needed. (ii) The effect of cocatalysts and supports on long-term catalyst stability and their role need to be researched. (iii) The OxHs and their effectiveness in reaction environment need to be stabilized. The main focus of studies on OxH catalysts should be on developing economically viable, highly stable, and environmentally friendly catalysts with large numbers of active sites, corrosive resistance, high electronic conductivity, and excellent electrocatalytic activity for the water-splitting reaction. The introduction of OxH-based materials to generate composites has been well established as a promising strategy for effectively increasing intrinsic activity in the water-splitting reaction process. We hope that this study sets the basis for the rational advancement of even more active and stable OxH electrocatalysts for this valuable water-splitting reaction. Furthermore, the challenges include the dependence of the Ni and Fe oxidation state on the water-splitting reaction at different transition metal contents. In general, most of the water-splitting reactions are carried out under alkaline conditions. So, a main challenge is to develop an active electrocatalyst working under all pH conditions. Some of the discussed electrocatalysts have several drawbacks in water-splitting reactions, including poor catalytic sites on the surface, fast electron–hole recombination rates, and a wide band-gap range that allows only UV light.

## Figures and Tables

**Table 1:** The EC performance of recently reported OxH-based electrocatalysts for water splitting, OER, and ORR.

Electrocatalyst	Medium (M)	Over Potential (V)	Tafel Slope (mV dec^−1^)	Current Density (mA cm^−2^)	Specific Capacity (mAh g^−1^)	Stability (Cycles)	Ref.
^a^ FeCo_2_O_x_(OH)_y_/GO	0.1 NaOH	0.305	43.7	10	−	−	[26]
^b^ 3D-FeCoOOH/CC	1 KOH	0.259	34.9	10	−	−	[27]
^c^ Spindle-FON/CC	-	0.216	73.4	10	−	−	[28]
^d^ NiFeOOH(S, Se)	1 KOH	0.195	31.99	10	−	−	[29]
^e^ FeCoNi(S)	1 KOH	0.293	−	500	−	2000 h	[30]
^f^ γ-FeOOH/N,S-C	0.1 NaOH	0.123	79.6	92	740	5000	[31]
^g^ RuO_2_/MnOOH/C	1 LiPF_6_	−	−	0.2	1000	100	[32]
^h^ γ-NiOOH	1 KOH	0.81	328	12	−	133	[33]
^i^ CoFe-OH@FeOOH	1 KOH	0.200	48	10	−	500 s	[34]
^j^ 3D CoFe-OOH@Co_2_P/NF	1 KOH	0.199	39	30	−	24 h	[35]

^a^ Iron cobalt oxyhydroxide based graphene oxide composite; ^b^ iron-induced 3D nanoporous iron–cobalt oxyhydroxide on carbon cloth; ^c^ spindle-shaped ferric oxide nanoparticle/carbon cloth; ^d^ sulfur- and selenium-based nickel iron oxyhydroxide; ^e^ trimetallic oxyhydroxides; ^f^ nitrogen and sulfur co-doped iron oxyhydroxide on carbon nanosheets; ^g^ ruthenium oxide nanoparticle decorated manganese oxyhydroxide on carbon; ^h^ nickel oxyhydroxides; ^i^ cobalt iron hydroxyl based iron oxyhydroxide nanocomposite; ^j^ cobalt iron oxyhydroxide based cobalt phosphide on nickel foam (CFCN) composite.

## Data Availability

Not applicable.

## Abbreviations

OER	Oxygen evolution reaction
HER	Hydrogen evolution reaction
OxHs	Oxyhydroxides
HT	Hydrothermal
ORR	Oxygen reduction reaction
DFT	Density functional theory
EC	Electrochemical

## References

[1] Zhou H., Li H., Li L., Liu T., Chen G., Zhu Y., Zhou L., Huang H. (2022). Structural composite energy storage devices—A review. Mater. Today Energy.

[2] Zhang R., Cheng L.Z., Wang Z., Kong F.Y., Tsegazab Y., Lv W.X., Wang W. (2020). Ni_3_S_2_Co_9_S_8_ heterostructure nanowire supported on Ni foam as highly efficient and stable electrocatalyst for oxygen evolution reaction. Appl. Surf. Sci..

[3] Roger I., Shipman M.A., Symes M.D. (2017). Earth abundant catalyst for electrochemical and photo electrochemical water splitting. Nat. Rev. Chem..

[4] Wang H., Ren X., Chen J., Xu W., He Q., Wang H., Zhan F., Chen L. (2023). Recent advances of emerging oxyhydroxide for electrochemical energy storage applications. J. Power Sources.

[5] Yu C., Lu J., Luo L., Xu F., Shen P.K., Tsiakaras P., Yin S. (2019). Bifunctional catalysts for overall water splitting: CoNi oxyhydroxide nanosheets electrodeposited on titanium sheets. Electrochimi. Acta.

[6] Lee S., Chu Y.C., Bai L., Chen H.M., Hu X. (2023). Operando identification of a side-on nickel superoxide intermediate and the mechanism of oxygen evolution on nickel oxyhydroxide. Chem. Catal..

[7] Guo B., Huo H., Zhuang Q., Ren X., Wen X., Yang B., Huang X., Chang Q., Li S. (2023). Iron oxyhydroxide: Structure and applications in electrocatalytic oxygen evolution reaction. Adv. Funct. Mater..

[8] Kim T.W., Choi K.S. (2014). Nanoporous photoanodes with dual-layer oxygen evolution catalyst for solar water-splitting. Science.

[9] Landon J., Demeter E., Inoglu N., Keturakis C., Wachs I.E., Vasic R., Frenkel A.I., Kitchin J.R. (2012). Spectroscopic characterization of mixed Fe-Ni oxide electrocatalysts for the oxygen evolution reaction in alkaline electrolytes. ACS Catat..

[10] Sorochkina K., Smotraiev R., Chepurna I. (2015). Zirconium and aluminium oxyhydroxides particles formation during sol-gel process. Colloids Surf. A Phys. Eng. Aspects.

[11] Mikhaylov V.I., Maslennikova T.P., Ugolkov U.L., Krivoshapkin P.V. (2016). Hydrothermal synthesis, characterization and sorption properties of AC/Fe oxide-oxyhydroxide composite powders. Adv. Power Technol..

[12] Motlagh M.M.K., Youzbashi A.A., Hashemzadeh F., Sabaghadeh L. (2013). Structural properties of nickel hydroxides/oxyhydroxide and oxide nanoparticles obtained by microwave assisted oxidation technique. Powder Technol..

[13] Wong K.C., Goh P.S., Suzaimi N.D., Ahmad N.A., Lim J.W., Ismail A.F. (2023). Copper catalyzed FeOOH template method for accelerated fabrication of ultraporous membranes used in microalgae dewatering. Chem. Eng. J..

[14] Xue Z., Wang Y., Yang M., Wang T., Zhu H., Rui Y., Wu S., An W. (2022). In-situ construction of electrodeposited polyaniline/nickel-iron oxyhydroxide stabilized on nickel foam for efficient oxygen evolution reaction at high current densities. Int. J. Hydrogen Energy.

[15] Fan H., Song J., Bai L., Wang Y., Jin Y., Liu S., Xie X., Zheng W., Liu W. (2023). Zn substituted hydroxide/oxyhydroxide heterostructure activates proton conduction. Energy Storage Mater..

[16] Lawrence M.J., Kolodziej A., Rodriquez P. (2018). Controllable synthesis of nanostructured metal oxide and oxyhydroxide materials via electrochemical methods. Curr. Opin. Electrochem..

[17] Miao Y., Quyang R., Zhou S., Xu L., Yang Z., Xiao M., Quyang R. (2014). Electrocatalysis and electroanalysis of nickel, its oxides, hydroxides and oxyhydroxides toward small molecules. Biosens. Bioelectron..

[18] Fleischman M., Korinek K., Pletcher D. (1971). The oxidation of organic compounds at a nickel anode in alkaline solutions. J. Electroanal. Chem. Interfacial Electrochem..

[19] Chen Z., Wang X., Kebler S., Fan O., Huang M., Colfen H. (2022). Synthesis of hierarchical transition metal oxyhydroxides in aqueous solution at ambient temperature and their application as OER electrocatalysts. J. Energy Chem..

[20] Mallick L., Rajput A., Adak M.K., Kundu A., Choudhary P., Chakraborty B. (2022). γ-FeO (OH) with multiple surface terminations: Intrinsically active for the electrocatalytic oxygen evolution reaction. Dalton Trans..

[21] El Issmaeli Y., Lahrichi A., Kalanur S.S., Natarajan S.K., Pollet B.G. (2023). Recent advances and prospects of FeOOH-based electrode materials for supercapacitors. Batteries.

[22] Liang C., Zou P., Nairan A., Zhang Y., Liu J., Liu K., Hu S., Kang F., Fan H.J., Yang C. (2020). Exceptional performance of hierarchical Ni–Fe oxyhydroxide@ NiFe alloy nanowire array electrocatalysts for large current density water splitting. Energy. Environ. Sci..

[23] Saidin N.U., Choo T.F., Yunus R.M., Zali N.M., Kok K.Y., Wong W.Y., Lim K.L. (2023). One-pot gamma radiolysis synthesis of a graphene oxide supported cobalt oxyhydroxide electrocatalyst for oxygen reduction reaction. Radiat. Phys. Chem..

[24] Yue Y., Niu J., Yang C., Qin J., Zhang X., Liu R. (2023). The OER/ORR activities of copper oxyhydroxides series electrocatalysts. Mol. Catal..

[25] Hefnawy M.A., Fadlallah S.A., Shrif R.M.E., Medany S.S. (2021). Nickel-manganese double hydroxide mixed with reduced graphene oxide electrocatalyst for efficient ethylene glycol electrooxidation and hydrogen evolution reaction. Syn. Metals.

[26] Tai J., Bi Y., Ni C., Wu X., Koudama T.D., Cui S., Shen X., Chen X. (2023). Modulating interfacial charge density of FeCo oxyhydroxides via coupling with graphene oxide aero-gel for boosting oxygen evolution reaction. J. Alloy. Compd..

[27] Chen G., Du J., Wang X., Shi X., Wang Z., Liang H.P. (2019). Iron-induced 3D nanoporous iron-cobalt oxyhydroxide on carbon cloth as a highly efficient electrode for oxygen evolution reaction. Chin. J. Catal..

[28] Lee S., Kim M.C., Lang A.R., Sohn J.I., Park J.B., Lee Y.W. (2022). Spindle-shape ferric oxyhydroxides with nano-sized grains for efficient oxygen evolution reaction and supercapacitors. Appl. Surf. Sci..

[29] Zhao C., Li J., Yang M., Chen J., Xie F., Wang N., Jin Y., Yu X., Meng H. (2022). S/Se dural-doping promotes the formation of active Ni/Fe oxyhydroxide for oxygen evolution reaction of (sea) water splitting. Int. J. Hydrogen Energy.

[30] Chen L., Wang Y., Zhao X., Wang Y., Li Q., Wang Q., Tang Y., Lei Y. (2022). Trimetallic oxyhydroxides as active sites for large-current density alkaline oxygen evolution and overall water splitting. J. Mater. Sci. Technol..

[31] Jia N., Liu J., Liu Y., Wang L., Chen P., An Z., Chen X., Chen Y. (2020). In situ conversion of iron sulfide (FeS) to iron oxyhydroxide (γ-FeOOH) on N,S Co-doped porous carbon nanosheets: An efficient electrocatalyst for the oxygen reduction reaction and zinc-air batteries. J. Colloids Interface Sci..

[32] Kim G.P., Lim D., Park I., Park H., Shim S.E., Baeck S.H. (2016). RuO_2_ nanoparticles decorated MnOOH/C as efficient bi-functional electrocatalysts for lithium-air battery cathode with long-cycling stability. J. Power Sources.

[33] Putra R.P., Rachman I.B., Horino H., Rzeznicka I.I. (2022). Bi-functional catalytic activity of γ-NiOOH toward oxygen reduction and oxygen evolution reactions in alkaline solutions. Oxygen.

[34] Babar P., Patil K., Mohmood J., Kim S., Kim J.H., Yavuz C.T. (2022). Low-overpotential overall water splitting by a co-operative interface of cobalt-iron hydroxide and iron oxyhydroxide. Cell Rep. Phys. Sci..

[35] Duan R., Li Y., Gong S., Tong Y., Li Z., Qi W. (2020). Hierarchical CoFe oxyhydroxides nanosheets and Co_2_P nanoparticles grown on Ni foam for overall water splitting. Electrochim. Acta.

[36] Tee S.Y., Win K.Y., Teo W.S., Koh L.D., Teng C.P., Han M.Y. (2017). Recent progress in energy-driven water splitting. Adv. Sci..

[37] Anantharaj S., Sugime H., Noda S. (2021). Surface amorphized nickel hydroxyl sulphise for efficient hydrogen evolution reaction in alkaline medium. Chem. Eng. J..

[38] Huang Y., Wei G., He J., An C., Hu M., Shu M., Zhu J., Yao S., Xi W., Si R. (2020). Interfacial electronic interaction of automatically dispersed IrCl_x_ on ultrathin Co(OH)_2_/CNTs for efficient electrocatalytic water oxidation. Appl. Catal. B Environ..

[39] Ramírez A.M., Heidari S., Vergara A., Aguilera M.V., Preuss P., Camarada M.B., Fischer A. (2023). Rhenium-Based Electrocatalysts for Water Splitting. ACS Mater. Au.

[40] Zhao J.W., Li C.F., Shi Z.X., Guan J.L., Li G.R. (2020). Boosting lattice oxygen oxidation of perovskite to efficiently catalyze oxygen evolution reaction by FeOOH decoration. Research.

[41] Peng X., Pi C., Zhang X., Li S., Huo K., Chu P.K. (2019). Recent progress of transition metal nitrides for efficient electrocatalytic water splitting. Sustain. Energy Fuels.

[42] Shi H., Liang H., Ming F., Wang Z. (2017). Efficient overall water splitting electrocatalysis using lepidocrocite VOOH hollow nanospheres. Angew. Chem. Int. Ed..

[43] Zhang P., Gong L., Tan Y. (2021). Ni_1− 2x_ Mo_x_Se nanowires@ ammonium nickel phosphate–MoO_x_ heterostructures as a high performance electrocatalyst for water splitting. Sustain. Energy. Fuels.

[44] Kim Y.K., Kim I.H., Jo Y.H., Lee J.S. (2019). Precipitating metal nitrate deposition of amorphous metal oxyhydroxide electrodes containing Ni, Fe and Co for electrocatalytic water oxidation. ACS Catal..

[45] Badreldin A., Nabeech A., Youssef E., Mubarak N., Elsayed H., Mohsen R., Ahmed F., Wubulikasimu Y., Elsaid K., Wahab A.A. (2021). Adapting early transition metal and nonmetallic dopants on CoFe oxyhydroxides for enhanced alkaline and neutral pH saline water oxidation. ACS Appl. Energy Mater..

[46] Zhang Z., He B., Chen B., Wang H., Wang R., Zho L., Gong Y. (2018). Boosting overall water splitting via FeOOH nanoflakes decorated PrBa_0.5_Sr_0.5_Co_2_O_5+δ_ nanorods. ACS Appl. Mater. Interfaces.

[47] Tsai F.T., Wang H.C., Ke C.H., Liaw W.F. (2018). FeCo/FeCoP_x_O_y_(OH)_2_ as bifunctional electrodeposited film electrodes for overall water splitting. ACS Appl. Energy Mater..

[48] Kou Z., Yu Y., Liu X., Gao X., Zheng L., Zou H., Pang Y., Wang Z., Pan Z., He J. (2020). Potential dependent phase transition and Mo enriching surface reconstruction of γ-CoOOH in heterostructured Co-Mo_2_C pre-catalyst enable water oxidation. ACS Catal..

[49] Wang P., Lin Y., Xu Q., Wan L., Xu Z., Wang B. (2022). The FeOOH decorated Fe-doped nickel selenide hierarchical array for high-performance water oxidation. Ind. Eng. Chem. Res..

[50] Moi C.T., Sahu A., Qureshi M. (2023). Tapping the potential of high-valent Mo and W metal centres for dynamic electronic structure in multi-metallic FeVO(OH)Ni(OH)_2_ for ultrastable and efficient overall water splitting. ACS Appl. Mater. Interfaces.

[51] Vazhayil A., Vazhayal L., Thomas J., Ashok S., Thomas N. (2021). A comprehensive review on the recent developments in transition metal based electrocatalysts for oxygen evolution reaction. Appl. Surf. Sci. Adv..

[52] Ghouri Z.K., Elsaid K., Nasef M.M., Ahmed S., Badreldin A., Wahab A.A. (2022). Cooperative electrocatalytic effect of Pd and Ce alloys nanoparticles in PdCe@CNWs electrodes for oxygen evolution reaction (OER). Mol. Catal..

[53] Pal S., Jana S., Kumar A., Parakash R.R. (2022). Enhanced OER properties from nanocomposites of Co_3_O_4_ and MOF derived N/S/Zn-doped porous carbon. Electrochim. Acta.

[54] Zhang R., Liu W., Zhang F.M., Yang Z.D., Zhang G., Zeng X.C. (2023). CoF-C_4_N nanosheets with uniformly anchored single metal sites for electrocatalytic OER: From theoretical screening to target synthesis. Appl. Catal. B Environ..

[55] Dionigi F., Strasser P. (2016). NiFe-based (oxy)hydroxide catalysts for oxygen evolution reaction in non-acidic electrolytes. Adv. Energy Mater..

[56] Sun X., Guan X., Feng H., Zheng D., Tian W., Li C., Li C., Yan M., Yao Y. (2021). Enhanced activity promoted by amorphous metal oxyhydroxides on CeO_2_ for alkaline oxygen evolution reaction. J. Colloid Interface Sci..

[57] Park J.H., Woo S., Lee J., Jung H.Y., Ro J.C., Park C., Lim B., Suh S.J. (2021). Facile modified polyol synthesis of FeCo nanoparticles with oxyhydroxide surface layer as efficient oxygen evolution reaction electrocatalysis. Int. J. Hydrog. Energy.

[58] Kim B., Kabraz M.K., Lee J., Choi C., Baik H., Jung Y., Oh H.S., Choi S.I., Lee K. (2021). Vertically-crystalline Fe-doped β-Ni oxyhydroxides for highly active and stable oxygen evolution reaction. Matter.

[59] Shao B., Pang W., Tan X.Q., Tang C., Deng Y., Huang D., Huang J. (2019). Rapid growth of amorphous cobalt-iron oxyhydroxide nanosheet arrays on to iron foam: Highly efficient and low-cost catalyst for oxygen evolution. J. Electroanal. Chem..

[60] Wang J., Zhang W., Zheng Z., Liu J., Yu C., Chen Y., Ma K. (2019). Dendric core-shell Ni@Ni(Fe)OOH metal/metal oxyhydroxide electrode for efficient oxygen evolution reaction. Appl. Surf. Sci..

[61] Guio C.G.M., Liardet L., Hu X. (2016). Oxidatively electrodeposited thin-film transition metal (oxy)hydroxides as oxygen evolution catalysts. J. Am. Chem. Soc..

[62] Li Y., Lin X., Du J. (2021). Iron-facilitated transformation of mesoporous spinel nanosheets in to oxyhydroxide active species in the oxygen evolution reaction. Inorg. Chem..

[63] He Q., Xie H., Rehman Z., Wang C., Wan P., Jiang H., Chu W., Song L. (2018). Highly defective Fe-based oxyhydroxides from electrochemical reconstruction for efficient oxygen evolution catalysis. ACS Energy Lett..

[64] Shamraiz U., Badsha A., Raza B., Green I.R. (2020). First principle study on chromium substituted α-cobalt oxyhydroxides for efficient oxygen evolution reaction. ACS Appl. Energy Mater..

[65] Deshpande N.G., Kim D.S., Ahn C.H., Jung S.H., Kim Y.B., Lee H.S., Cho H.K. (2021). β-like FeOOH nanoswords activated by Ni foam and encapsulated by rGO towards high current densities durability and efficient oxygen evolution. ACS Appl. Mater. Interfaces.

[66] Niu S., Sun Y., Rakov D., Li Y., Ma Y., Chu J., Xu P. (2019). Stepwise electrochemical construction of FeOOH/Ni(OH)_2_ on Ni foam for enhanced electrocatalytic oxygen evolution. ACS Appl. Energy Mater..

[67] Zhang Y., You L., Liu Q., Li Y., Li T., Xue Z., Li G. (2021). Interfacial charge transfer in a hierarchical Ni_2_P/FeOOH heterojunction facilitates electrocatalytic oxygen evolution. ACS Appl. Mater. Interfaces.

[68] Anantharaja S., Noda S., Jothi V.R., Yi J.S., Driess M., Menezes P.W. (2021). Strategies and perspectives to catch the missing pieces in energy efficient hydrogen evolution reaction in alkaline media. Angew. Chem. Int. Ed..

[69] Cheng J., Wang D. (2022). 2D materials modulating layered double hydroxides for electrocatalytic water splitting. Chin. J. Catal..

[70] Liu Y., Ding M., Tian Y., Zhao G., Huang J., Xu X. (2023). In-situ growth of 3D hierarchical γ-FeOOH/Ni_3_S_2_ heterostructure as high performance electrocatalyst for overall water splitting. J. Colloid Interface Sci..

[71] Zhang K., Wang F., Li X., Wang S., Wang Y., Zha Q., Ni Y. (2023). Connected design of Ni-foam supported porous Ni and FeOOH/porous Ni electrocatalysis for overall water splitting in alkaline media. J. Alloy. Compd..

[72] Lv Q., Zhang W., She L., Ren W., Hou L., Fautrelle Y., Lu X., Yu X. (2022). Controlled direct electrodeposition of crystalline NiFe/amorphous NiFe (oxy)hydroxide on NiMo-alloy as a highly efficient bi-fucntional electrocatalyst for overall water splitting. Chem. Eng. J..

[73] Mu W., Bao D., Chang C. (2022). Growth of nickel vacancy NiFe-LDHs on Ni(OH)_2_ nanosheets as highly efficient bi-functional electrocatalyst for overall water splitting. Int. J. Hydrog. Energy.

[74] Liu H., Cao S., Zhang J., Liu S., Chen C., Zhang Y., Wei S., Wang Z., Lu X. (2021). Facile control of surface reconstruction with Co^2+^ or Co^3+^-rich (oxy)hydroxide surface on ZnCo phosphate for large-current-density hydrogen evolution in alkali. Mater. Today Phys..

[75] Li X., Hu Q., Wang H., Chen M., Hao X., Ma Y., Liu J., Tang K., Abudula A., Guan G. (2021). Charge induced crystal distortion and morphology remodeling: Formation of Mn-CoP nanowire@Mn-COOH nanosheet electrocatalyst with rich edge dislocation defects. Appl. Catal. B Environ..

[76] Anantharaj S., Subhashini E., Swaathini K.C., Amarnath T.C., Chatterjee S., Karthick K., Kundu S. (2019). Respective influence of stoichiomery and NiOOH formation in hydrogen and oxygen evolution reactions of nickel selenides. Appl. Surf. Sci..

[77] Tao J., Zhang Y., Wang S., Ge W., Hu F., Yan X., Hao L., Zuo Z., Yang X. (2019). Activating three-dimensional networks on Fe@Ni nanofibers via fast surface modification for efficient overall water splitting. ACS Appl. Mater. Interfaces.

[78] Hu J., Ou Y., Gao D., Zhang Y., Xiao P. (2018). FeCo_2_S_4_ nanosheet arrays supported on Ni foam: An efficient and durable bi-fuctional electrocatalyst for overall water splitting. ACS Sustain. Chem. Eng..

[79] Yan Y., Liu J., Huang K., Qi J., Qiao L., Zheng X., Cai W. (2021). A fast micro–nano liquid layer induced construction of scaled-up oxyhydroxide based electrocatalysts for alkaline water splitting. J. Mater. Chem. A.

[80] She S., Zhu Y., Tahini H.A., Wu X., Guan D., Chen Y., Dai J., Chen Y., Tang W., Smith S.C. (2020). Efficient water splitting actualized through an electrochemistry induced heterostructured antiperovskite/(oxy) hydroxide hybrid. Small.

[81] Liu Y., Zhou D., Deng T., He G., Chen A., Sun X., Yang Y., Miao P. (2021). Research progress of oxygen evolution reaction catalysts for electrochemical water splitting. ChemSusChem.

[82] Chen Y., Rui K., Zhu J., Dou S.X., Sun W. (2019). Recent progress on nickel-based oxide/(oxy) hydroxide electrocatalysts for the oxygen evolution reaction. Chem.–A Eur. J..

[83] Zhu W., Chen W., Yu H., Zeng Y., Ming F., Liang H., Wang Z. (2020). NiCo/NiCo–OH and NiFe/NiFe–OH core shell nanostructures for water splitting electrocatalysis at large currents. Appl. Catal. B: Environ..

[84] Yan Y., Huang K., Lin J., Yang T., Wang P., Qiao L., Cai W., Zheng X. (2023). Charge redistribution in FeOOH nanoarray by ecological oxygen-reduction deposition for boosting electrocatalytic water oxidation. Appl. Catal. B Environ..

[85] Ge Z., Wang F., Guo J., Ma J., Yu C., Zhong A., Xie Y. (2021). Low-cost and multi-level structured NiFeMn alloy@NiFeMn oxyhydroxide electrocatalysts for highly efficient overall water splitting. Inorg. Chem. Front..

[86] Gao M.Y., Sun C.B., Lei H., Zeng J.R., Zhang Q.B. (2018). Nitrate-induced and in situ electrochemical activation synthesis of oxygen deficiencies-rich nickel/nickel (oxy) hydroxide hybrid films for enhanced electrocatalytic water splitting. Nanoscale.

[87] Mathi S., Jayabharathi J. (2020). Enhanced stability and ultrahigh activity of amorphous ripple nanostructured Ni-doped Fe oxyhydroxide electrode toward synergetic electrocatalytic water splitting. RSC Adv..

[88] Poudel M.B., Kim H.J. (2022). Confinement of Zn-Mg-Al-layered double hydroxide and α-Fe_2_O_3_ nanorods on hollow porous carbon nanofibers: A free-standing electrode for solid-state symmetric supercapacitors. Chem. Eng. J..

[89] Poudel M.B., Logeshwaran N., Kim A.R., Karthikeyan S.C., Vijayapradeep S., Yoo D.J. (2023). Integrated core-shell assembly of Ni_3_S_2_ nanowires and CoMoP nanosheets as highly efficient bifunctional electrocatalysts for overall water splitting. J. Alloy. Compd..

[90] Wang N., Xu A., Ou P., Hung S.F., Ozden A., Lu Y.R., Abed J., Wang Z., Yan Y., Sun M.J. (2021). Boride-derived oxygen-evolution catalysts. Nat. Commun..

[91] Wu Z.P., Zhang H., Zuo S., Wang Y., Zhang S.L., Zhang J., Zang S.Q., Lou X.W. (2021). Manipulating the Local Coordination and Electronic Structures for Efficient Electrocatalytic Oxygen Evolution. Adv. Mater..

[92] Yang X., Zhao F., Yeh Y.W., Selinsky R.S., Chen Z., Yao N., Tully C.G., Ju Y., Koel B.E. (2019). Nitrogen-plasma treated hafnium oxyhydroxide as an efficient acid-stable electrocatalyst for hydrogen evolution and oxidation reactions. Nat. Commun..

[93] Liu H., Jang H., Wang Y., Kim M.G., Li H., Qin Q., Liu X., Cho J. (2022). IrO_2_/LiLa_2_ IrO_6_ as a robust electrocatalyst for the oxygen evolution reaction in acidic media. J. Mater. Chem. A.

[94] Rodney J.D., Deepapriya S., Das S.J., Robinson M.C., Perumal S., Katlakunta S., Sivakumar P., Jung H., Raj C.J. (2022). Boosting overall electrochemical water splitting via rare earth doped cupric oxide nanoparticles obtained by co-precipitation technique. J. Alloy. Compd..

[95] Li Y., Zhang X., Wu Z., Sheng H., Li C., Li H., Cao L., Dong B. (2021). Coupling porous Ni doped LaFeO_3_ nanoparticles with amorphous FeOOH nanosheets yields an interfacial electrocatalyst for electrocatalytic oxygen evolution. J. Mater. Chem. A..

[96] Nisa M.U., Gassoumi A., Alharbi F.F., Aman S., Manzoor S. (2023). Understanding the spatial configurations of Sm_2_O_3_ in ZnO surface-loaded or embedded for the electrocatalytic oxygen evolution reaction. J. Sol-Gel Sci. Technol..

[97] Swathi S., Yuvakkumar R., Ravi G., Al-Sehemi A.G., Velauthapillai D. (2022). Rare earth metal (Sm)-doped NiMnO_3_ nanostructures for highly competent alkaline oxygen evolution reaction. Nanoscale Adv..

[98] Al-Naggar A.H., Shinde N.M., Kim J.S., Mane R.S. (2023). Water splitting performance of metal and non-metal-doped transition metal oxide electrocatalysts. Coord. Chem. Rev..

[99] Rabia M., Hadia N.M.A., Farid O.M., Abdelazeez A.A.A., Mohamed S.H., Shaban M. (2022). Poly(m-toluidine)/rolled graphene oxide nanocomposite photocathode for hydrogen generation from wastewater. Inter. J. Energy Res..

[100] Alshehri S.M., Ahmed J., Khan A., Naushad M., Ahamad T. (2018). Bifunctional electrocatalysts (Co_9_S_8_@NSC) derived from a polymer-metal complex for the oxygen reduction and oxygen evolution reactions. ChemElectroChem.

[101] Yuki S., Damian K., Yoshitaka A., Hiroki H. (2020). Long-term durability of platelet-type carbon nanofibers for OER and ORR in highly alkaline media. Appl. Catal. A Gen..

[102] Gumaa A., El-Nagar I.A., Fetyan C.R. (2017). One-pot Synthesis of a High performance chitosan-nickel oxyhydroxide nanocomposite for glucose fuel cell and electro-sensing applications. Appl. Catal. B Environ..

[103] Gaowei Z., Junrong Z., Jing Y., Chunyan Z., Peng W., Hongtao C., Yejun Q. (2021). Iron-facilitated surface reconstruction to in-situ generate nickel-iron oxyhydroxide on self-supported FeNi alloy fiber paper for efficient oxygen evolution reaction. Appl. Catal. B Environ..

[104] Lina T., Tingting F., Zhou C., Jianling T., Hongquan G., Yang L., Jianhui L.b., Yifei S. (2021). Binary-dopant promoted lattice oxygen participation in OER on cobaltate electrocatalyst. Chem. Eng. J..

[105] Weichuan X., Litao Y., Lara T., Steven L., Meng Z., Hongmei L. (2018). Polymer-assisted chemical solution synthesis of La_0.8_Sr_0.2_MnO_3_-based perovskite with A-site deficiency and cobalt-doping for bi-functional oxygen catalyst in alkaline media. Electrochim. Acta.

[106] Digambar V., Radha A.V., Wolfgang S., Corina A., Sebastian W. (2020). Trivalent iron rich CoFe layered oxyhydroxides for electrochemical water oxidation. Electrochim. Acta.

[107] Junyang D., Qian S., Li Z., Xian W., Lulu C., Qipeng L., Ting T.L., Shaoming H. (2020). Thermal conversion of hollow nickel-organic framework into bimetallic FeNi_3_alloy embedded in carbon materials as efficient OER electrocatalyst. Electrochim. Acta.

[108] Xinqi H., Zihan L., Xingquan H., Lili C., Dong X.L. (2020). The construction of defective FeCo-LDHs by in-situ polyaniline curved strategy as a desirable bi-functional electrocatalyst for OER and HER. Inter. J. Hydrogen Energy.

[109] Pravin B., Komal P., Vijay K., Kuldeep G., Sambhaji P. (2021). Cost-effective and efficient water and urea oxidation catalysis using nickel-iron oxyhydroxide nanosheets synthesized by an ultrafast method. J. Colloid Interface Sci..

[110] Panel R.P.P., Budi R., Hideyuki H., Izabela I.R. (2022). *γ*-NiOOH electrocatalyst derived from a nickel dithiooxamide chelate polymer for oxygen evolution reaction in alkaline solutions. Catal. Today.

[111] Phu A., Rahman D., Joshua L., Zelio F., Anton T., Iolanda D.B., Alexander K. (2022). Understanding the activity and stability of flame-made Co_3_O_4_spinels: A route towards the scalable production of highly performing OER electrocatalysts. Chem. Eng. J..

[112] Badreldin A., Youssef K., Ghenymy A.E., Wubulikasimu Y., Ghouri Z.K., Elsaid K., Kumar D., Wahab A.A. (2022). Solution combustion synthesis of novel S,B-co-doped CoFe oxyhydroxides for the oxygen evolution reaction in saline water. ACS Omega.

[113] Chen F.Y., Wu Z.Y., Adler Z., Wang H. (2021). Stability challenges of electrocatalytic oxygen evolution reaction: From mechanistic understanding to reactor design. Joule.

[114] Zhao L., Faqiong Zhao F., Zeng B. (2013). Electrochemical determination of methyl parathion using a molecularly imprinted polymer-ionic liquid-graphene composite film coated electrode. Sens. Actuators B Chem..

[115] Mohammadi V., Rosen J., Gogotsi Y. (2021). The world of two-dimensional carbides and nitrides (Mxenes). Science.

[116] Naguib M., Kurtoglu M., Presser V., Lu J., Niu J., Heon M., Hultman L., Gogotsi Y., Barsoum M.W. (2011). Two-dimensional nanocrystals produced by exfoliation of Ti_3_AlC_2_. Adv. Mater..

[117] Alhabeb M., Maleski K., Anasori B., Lelyukh P., Clark L., Sin S., Gogotsi Y. (2017). Guidelines for synthesis and processing of two-dimensional titanium carbide (Ti_3_C_2_Tx MXene). Chem. Mater..

[118] Jun B.M., Kim S., Heo J., Park C.M., Her N., Jang M., Huang Y., Han J., Yoon Y. (2019). Review of MXenes as new nanomaterials for energy storage/delivery and selected environmental applications. Nano Res..

[119] Li Y., Zhu S., Wu E., Ding H., Lu J., Mu X., Chen L., Kuang Y., Chai Z., Huang Q. (2023). Nanolaminated ternary transition metal carbide (MAX phase)-derived core–shell structure electrocatalysts for hydrogen evolution and oxygen evolution reactions in alkaline electrolytes. J. Phys. Chem. Lett..

[120] Zou H., He B., Kuang P., Yu J., Fan K. (2018). Metal–organic framework-derived nickel-cobalt sulfide on ultrathin Mxene nanosheets for electrocatalytic oxygen evolution. ACS Appl. Mater. Interfaces.

[121] Loupias L., Boule R., Morais C., Mauchamp V., Guignard N. (2023). Mo_2_CT_x_ MXene supported nickel-iron alloy: An efficient and stable heterostructure to boost oxygen evolution reaction. 2D Maters..

[122] Han S., Chen Y., Hao Y., Xie Y., Xie D., Chen Y., Xiong Y. (2021). Multi-dimensional hierarchical CoS_2_@MXene as trifunctional electrocatalysts for zinc-air batteries and overall water splitting. Sci. China Mater..

[123] Li N., Han J., Yao K., Han M., Wang Z., Liu Y., Liu L., Liang H. (2022). Synergistic phosphorized NiFeCo and MXene interaction inspired the formation of high-valence metal sites for efficient oxygen evolution. J. Mater. Sci. Technol..

[124] Priamushko T., Guggenberger P., Mautner A., Lee J., Ryoo R., Kleitz F. (2022). Enhancing OER Activity of Ni/Co Oxides via Fe/Mn Substitution within Tailored Mesoporous Frameworks. ACS Appl. Energy Mater..

[125] Cheng X., Kim B.J., Fabbri E., Schmidt T.J. (2019). Co/Fe oxyhydroxides supported on perovskite oxides as oxygen evolution reaction catalyst systems. ACS Appl. Mater. Interfaces.

[126] Yuan J., Cheng X., Lei C., Yang B., Li Z., Luo K., Lam K.H.K., Lei L., Hou Y., Ostrikov K.L. (2021). Bimetallic Oxyhydroxides as a high-performance water oxidation electrocatalysts under industry relevant conditions. Engineering.

[127] Lu J., Hao W., Wu X., Shen X., Cui S., Shi W. (2023). Electronic modulation of the 3D architecture Ni-Fe oxyhydroxide anchored N-Doped carbon aerogel wit much improved OER activity. Gels.

[128] Wang Z., Wang Y., Zhang N., Ma L., Sun J., Yu C., Liu S., Jiang R. (2022). Highly efficient oxygen evolution catalysis achieved by NiFe oxyhydroxide clusters anchored on carbon black. J. Mater. Chem. A.

[129] Xu Z., Hao M., Liu X., Ma J., Wang L., Li C., Wang W. (2022). Co(OH)_2_ nanoflowers decorated a-NiMoO_4_ nanowires as bi-functional electrocatalysts for efficient overall water splitting. Catalysts.

[130] Zhou Y., Wang Z., Pan Z., Liu L., Xi J., Luo X., Shen Y. (2019). Exceptional performance of Hierarchical Ni-Fe (Hydr)oxide@NiCu electrocatalysts for water splitting. Adv. Mater..

[131] Lawley C., Tehhrani Z.P., Clark A.H., Safonova O.V., Dobeli M., Strocov V.N., Schmidt T.J., Lippert T., Nachigaal M., Pergolesi D. (2022). Protagonists and spectators during photocatalytic solar water splitting with SrTaOxNy oxynitride. J. Mater. Chem. A.

[132] Liu Y., Hu Y., Ma P., Li F., Yuan F., Wang S., Luo Y., Ma J. (2019). Amorphous CoFe double hydroxide decorated with N-doped CNTs for efficient electrochemical oxygen evolution. ChemSusChem.

[133] Zhang T., Han J., Tang T., Sun J., Guan J. (2023). Binder-free bifunctional SnFe sulfide/oxyhydroxide heterostructure electrocatalysts for overall water splitting. Int. J. Hydrogen Energy.

[134] Zhang R., Guo B., Pan L., Huang Z.F., Shi C., Zhang X., Zou J.J. (2023). Metal-oxoacid- mediated oxyhydroxide with proton acceptor to break adsorption energy scaling relation for efficient oxygen evolution. J. Energy Chem..

[135] Feng S., Yang L., Zhang Z., Li Q., Xu D. (2020). Au-decorated CoOOH nanoplate hierarchical hollow structure for Plasmon enhanced elctrocatalytic water oxidation. ACS Appl. Mater..

